# Freestanding and Flexible Interfacial Layer Enables Bottom-Up Zn Deposition Toward Dendrite-Free Aqueous Zn-Ion Batteries

**DOI:** 10.1007/s40820-022-00921-6

**Published:** 2022-09-01

**Authors:** Hangjun Ying, Pengfei Huang, Zhao Zhang, Shunlong Zhang, Qizhen Han, Zhihao Zhang, Jianli Wang, Wei-Qiang Han

**Affiliations:** School of Materials Science and Engineering, Zhejiang University, Hangzhou, 310027 People’s Republic of China

**Keywords:** Aqueous zinc-ion battery, Flexible interfacial layer, Dendrite inhibition, Bottom-up deposition, Moderate zincophilicity

## Abstract

Aqueous rechargeable zinc ion batteries are regarded as a competitive alternative to lithium-ion batteries because of their distinct advantages of high security, high energy density, low cost, and environmental friendliness. However, deep-seated problems including Zn dendrite and adverse side reactions severely impede the practical application. In this work, we proposed a freestanding Zn-electrolyte interfacial layer composed of multicapsular carbon fibers (MCFs) to regulate the plating/stripping behavior of Zn anodes. The versatile MCFs protective layer can uniformize the electric field and Zn^2+^ flux, meanwhile, reduce the deposition overpotentials, leading to high-quality and rapid Zn deposition kinetics. Furthermore, the bottom-up and uniform deposition of Zn on the Zn-MCFs interface endows long-term and high-capacity plating. Accordingly, the Zn@MCFs symmetric batteries can keep working up to 1500 h with 5 mAh cm^−2^. The feasibility of the MCFs interfacial layer is also convinced in Zn@MCFs||MnO_2_ batteries. Remarkably, the Zn@MCFs||α-MnO_2_ batteries deliver a high specific capacity of 236.1 mAh g^−1^ at 1 A g^−1^ with excellent stability, and maintain an exhilarating energy density of 154.3 Wh kg^−1^ at 33% depth of discharge in pouch batteries.

**Supplementary Information:**

The online version contains supplementary material available at 10.1007/s40820-022-00921-6.

## Highlights

- Freestanding multicapsular carbon fibers (MCFs) cloth was synthesized by electrospinning and applied as interfacial layer to regulate the plating/stripping behavior of Zn anodes.
- MCFs layer is supposed to uniformize the electric field and Zn^2+^ flux, and the moderate zincophilicity enables the bottom-up deposition of Zn on Zn@MCFs anode, thereby leading to high-quality and rapid Zn deposition kinetics.
- Superior electrochemical performance of Zn@MCFs is achieved in symmetrical, asymmetrical and Zn||MnO_2_ batteries, including long cycling life, high coulombic efficiency and excellent rate performance.

**Supplementary Information:**

The online version contains supplementary material available at 10.1007/s40820-022-00921-6.

## Graphical abstract

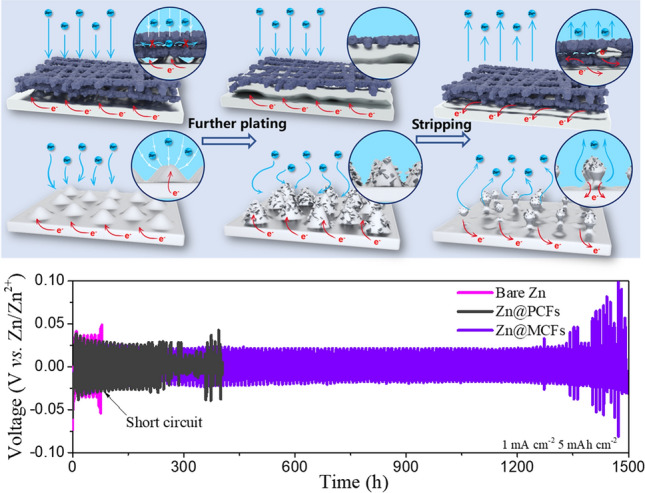

## Introduction

With the dramatic growth of electrochemical energy storage market, the battery safety issue has become a worldwide problem [1, 2]. One of the radical solutions is developing aqueous battery systems to eliminate the potential safety hazard from flammable organic electrolyte [3, 4]. Therefore, aqueous zinc ion batteries have attracted increasing attentions in recent years because of the merits including high safety, high capacity, easy fabrication, and so on [5–7]. The Zn metal exhibits high specific capacity of 820 mAh g^−1^ and low redox potential of − 0.76 V vs. SHE; however, the negative effects of dendrite growth and uncontrolled side reactions including hydrogen evolution and corrosion still severely hamper the practical application of Zn anode [8, 9]. Among these issues, the dendrite growth is the most harmful which may directly result in internal short-circuit.

Tremendous efforts have been paid to solve above-mentioned problems [5, 10, 11]. Recently, vast attention has been focused on the interface between Zn metal and electrolyte, which plays a crucial role in the electrochemical performance of Zn anode [12–14]. High-quality Zn plating relies on small nucleation overpotential and/or small plateau overpotential [5]. Based on these principles, researchers have designed diverse interfacial layers, which can be roughly categorized into insulating layer [15, 16], electron-oriented layer [17, 18], and ion-oriented layer [19–21]. Some inorganic porous materials can be used as insulating layer to isolate the zinc dendrites and guide uniform Zn^2+^ distribution over the surface of Zn anode. For example, porous CaCO_3_ coating can lead uniform plating of Zn, enabling a high capacity retention of 177 mAh g^−1^ after 1000 cycles in Zn||MnO_2_ aqueous battery [16]. In terms of electron-oriented layer, some electrically conductive materials are applied to homogenize the surface electric field, such as graphene and MXenes. For instance, Niu et al. decorated Ti_3_C_2_T_x-y_ film on Zn foil by in situ spontaneously reduction reaction. The MXene layer is demonstrated to endow the anode with a more uniform electric field and obviously lower voltage hysteresis, leading to excellent long-term cycling stability with 81% capacity retention after 500 cycles [22]. For ion-oriented layer, some polymer with ample polar groups and highly Zn^2+^-conductive inorganic substances are proved to be ideal choices. For example, by in situ formation of a highly Zn^2+^-conductive hopeite SEI layer, the Zn^2+^ transport kinetics can be significantly improved, enabling dendrite-free Zn depositing and controlled side reactions [23].

However, interfacial layer modification is still faced with some common issues, such as insufficient mechanical stability, poor flexibility, uncontrollable thickness, and so on. In order to solve these issues, herein, we inventively designed a freestanding interfacial layer composed of multicapsular carbon fibers (abbreviated as MCFs) by electrospinning technique. The electrically conductive MCFs layer is able to uniformize the surface charge distribution and improve Zn deposition kinetics, guiding formation of homogeneous Zn^2+^ flux. Furthermore, the multicapsular fibers show high electrolyte uptake, which can efficiently immobilize the electrolyte flow and inhibit the 2D diffusion of Zn^2+^ along the surface of the Zn foil. Remarkably, for the first time, we observed a novel bottom-up deposition of zinc on Zn@MCFs anode, which is different from the deposition modes on previously reported carbonaceous fibers [24, 25]. The bottom-up deposition of Zn on Zn@MCFs endows a flat and dense Zn electroplated layer. Therefore, the Zn@MCFs anode delivers long lifespan, high plating quantity, and high coulombic efficiency.

## Experimental and Calculation

### Preparation of MCFs, PAN-Derived Carbon Fibers (PCFs), Zn@MCFs and Zn@PCFs

The MCFs layer is synthesized by electrospinning technique followed by high-temperature pyrolysis. In briefly, ZIF-8 particles with average size around 100 nm were synthesized according to modified previous reported method [26, 27]. Firstly, 1.5 g zinc nitrate hexahydrate (Zn(NO_3_)_2_·6H_2_O, 99%, Aladdin) and 1.65 g 2-methylimidazole (C_4_H_6_N_2_, 98%, Aladdin) were, respectively, dissolved in 50 mL methanol (CH_3_OH, ≥ 99.9%, Aladdin). After stirring for a while, the former solution was poured into the later solution, and kept stirring vigorously for 10 min before aged for 10 h under room temperature. The ZIF-8 powder was collected by centrifugation and washed several times before vacuum drying at 80 °C overnight. For the preparation of MCFs cloth, 0.65 g ZIF-8 powder was dispersed in 5.5 mL N, N-dimethylformamide (DMF, C_3_H_7_NO, ≥ 99.5%, Sinopharm). Afterward, 0.216 g polymethyl methacrylate (PMMA, MW = 35,000, RG, Sinopharm) and 0.65 g polyacrylonitrile (PAN, MW = 85,000, RG, Aladdin) were added; then the mixture was stirred overnight at 60 °C to obtained uniform spinning suspension. The electrospinning was carried out with a positive voltage of 15 kV and a negative voltage of 2 kV, with a collecting distance of 15 cm. To obtain the MCFs, the precursor cloth was peeled off from the collector and treated at 250 °C under air for 3 h followed by carbonization at 700 °C under argon for 1.5 h. PAN-derived carbon fiber (abbreviated as PCFs) cloth is prepared by the same method, except addition of ZIF-8 template.

For preparing Zn@MCFs and Zn@PCFs anodes, the cleaned Zn foil was wetted by a small amount of electrolyte first. Afterward, the MCFs membrane was laid onto the Zn foil carefully to avoid wrinkles. The MCFs can be sticked firmly to the surface of Zn foil due to electrolyte surface tension. Then, the MCFs coated Zn foil was cut into small wafers or rectangular electrodes.

### Preparation of Needlelike α-MnO_2_

α-MnO_2_ nanoneedles were prepared according to previous report [28]. In typically, 0.5 g manganese sulfate monohydrate (MnSO_4_·H_2_O, 99%, Aladdin) and 2 mL sulfuric acid solution (H_2_SO_4_, 0.5 M, Sinopharm) were added into 60 mL deionized water, and stirred to dissolve. Then, 20 mL potassium permanganate solution (KMnO_4_, 0.1 M, Sinopharm) was added into above mixture and stirred for 1 h before sonicated for another 1 h. Afterward, the mixture was heated at 120 °C for 12 h in a Teflon-lined autoclave. The final product was obtained by centrifugation, washing and freeze-drying.

### Assembly of Symmetrical, Asymmetrical and Zn||MnO_2_ Cells

The symmetrical, asymmetrical and Zn||MnO_2_ batteries were assembled using 2032-type coin cell. For symmetrical and asymmetrical cells, bare Zn and coated Zn slices (diameter: 14 mm) were used as electrodes; 2 M zinc sulfate (ZnSO_4_, 99.5%, Aladdin) and glassfiber (Whatman 1823–110) were used as electrolyte and separator. For Zn||MnO_2_ coin and pouch cells, the cathodes were prepared by mixing MnO_2_ powder, multiwalled carbon nanotubes (MWCNTs, Aladdin) and polyvinylidene fluoride binder (PVDF, MW = 400,000, Aladdin) with a weight ratio of 6:3:1. The slurry was cast onto titanium mesh and dried at 80 °C overnight in vacuum. The loading mass of the cathode (MnO_2_) is around 1.0 mg cm^−2^ for coin cells and around 9.6 mg cm^−2^ for pouch cells. 100 μm thick Zn slices were used as counter electrode in coin cells and 10 μm thick Zn slices were used in pouch cells with a lean-Zn state. 2 M ZnSO_4_ + 0.1 M MnSO_4_ and glassfiber were applied as electrolyte and separator. Pouch cells with size of 2 × 8 and 2 × 3 cm^2^ were assembled. In detail, the well-cut electrodes were welded with nickel (on anode) and aluminum (on cathode) lugs first. Afterward, the anode and cathode were separated by glassfiber and neatly arranged in an aluminum-plastic bag with three sides pre-sealed. An appropriate amount of electrolyte (150 μm per square centimeter of anode) was added to the cell along the glassfiber. Finally, the last side was sealed by vacuum sealing to obtain pouch cells. In order to generate enough voltage to light the LEDs, the 2 × 8 cm^2^ pouch cells have two layers with internal series construction. The optical images of electrodes and 2 × 8 cm^2^ pouch cells are displayed in Fig. S37a-b. All batteries were assembled in the open atmosphere.

### Electrochemical Measurements

The Zn plating/stripping and charging/discharging tests were performed in a battery test system (Land, CT2001A). Cyclic voltammetry (CV), chronoamperometry (CA) and electrochemical impedance (EIS) spectra were carried out in a cell test system (Solartron analytical 1470E, 1400). The energy density of pouch cells can be calculated according to the following equation:

1 $$E = \frac{{m_{{{\text{cathode}}}} \times \int_{0}^{C} {{\text{discharge}}} \;\left( {C \times V} \right) \times 1000}}{{m_{{{\text{total}}}} }}$$

where ${\text{m}}_{\text{cathode}}$ (g) is the mass of cathode (MnO_2_), ${\text{C}}$ (Ah g^−1^) is the discharge capacity, V (V) is the voltage, and ${\text{m}}_{\text{total}}$ (g) is the total mass of the electrodes (Zn foil and MnO_2_). The integral part can be calculated according to the shaded area as shown in Fig. S39.

### Materials Characterizations

X-ray diffraction (XRD) spectra were collected in an x-ray diffractometer (Rigaku, miniflex 600). Morphology characterizations were carried out in a scanning electron microscopy (SEM, Hitachi, SU8010) and transmission electron microscopy (TEM, JEOL, JEM2100F). In situ observation of dendrite growth was implemented in an optical microscope (Sunny instruments, SOPTOP RX50M). Tensile test was carried out in an electronic universal test machine (Suns, UTM2102). Instantaneous contact angles were collected form contact angle surface tension measuring instrument (Dropmeter 100P). Fourier transform infrared spectroscopy (FTIR) was tested using a Fourier infrared spectrometer (Thermo Nicolet, 6700). X-ray photoelectron spectroscopy (XPS) was performed in an X-ray photoelectron spectrometer (Thermo Scientific, K-Alpha).

### Theoretical Calculation and Simulation

Electric field and ion concentration distribution on the surface of bare Zn and MCFs coated Zn were simulated based on simplified models. In the model, the length of the electrodes was 14 μm, and the thickness of electrolyte (electrode spacing) was 12 μm. The potential difference was 100 mV between the electrodes. The Zn protuberances on the surface showed the shape of cone with a diameter of 200 nm and height of 300 nm. The thickness of MCFs layer was 300 nm with a porosity of 85%. In addition, the ionic conductivity of electrolyte was 5 S m^−1^ (2 M ZnSO_4_), the electrical conductivity of Zn foil and MCFs layer were 1.67 × 10^7^ and 5 × 10^2^ S m^−1^, respectively. Of note, the model was established under approximate conditions and cannot fully reflect the actual state.

Density functional theory (DFT) calculations were performed based on the pseudopotential plane wave (PPW) method using quantum espresso (QE) [29, 30]. The Perdew–Bueke–Ernzerhof (PBE) function was used to describe exchange–correlation effects of electrons [31]. The projected augmented wave (PAW) potentials were chosen to describe the ionic cores and valence electrons were considered using a plane wave basis set with a kinetic energy cutoff of 500 eV [32, 33]. The Brillouin-zone sampling were conducted using Monkhorst–Pack (MP) grids of special points with a separation of 0.04 Å^−1^ [34]. The convergence criterion for the electronic self-consistent field (SCF) loop was set to 1 × 10^–5^ eV atom^−1^. The surface atomic configuration models were constructed according to the FTIR and XPS results. For fair comparison, the absorption of Zn onto Zn (002) and Zn (101) planes was calculated, and three adsorption sites of Zn clusters were chosen to confirm the preferred structure of MCFs. To avoid interactions, an extra vacuum zone of 12 Å along z direction was added when we simulated the surface of Zn crystal. All structures were optimized to reach their most stable configurations. The atomic structures were fully relaxed until the residual forces were below 0.05 eV Å^−1^.

## Results and Discussions

**Fig. 1:**
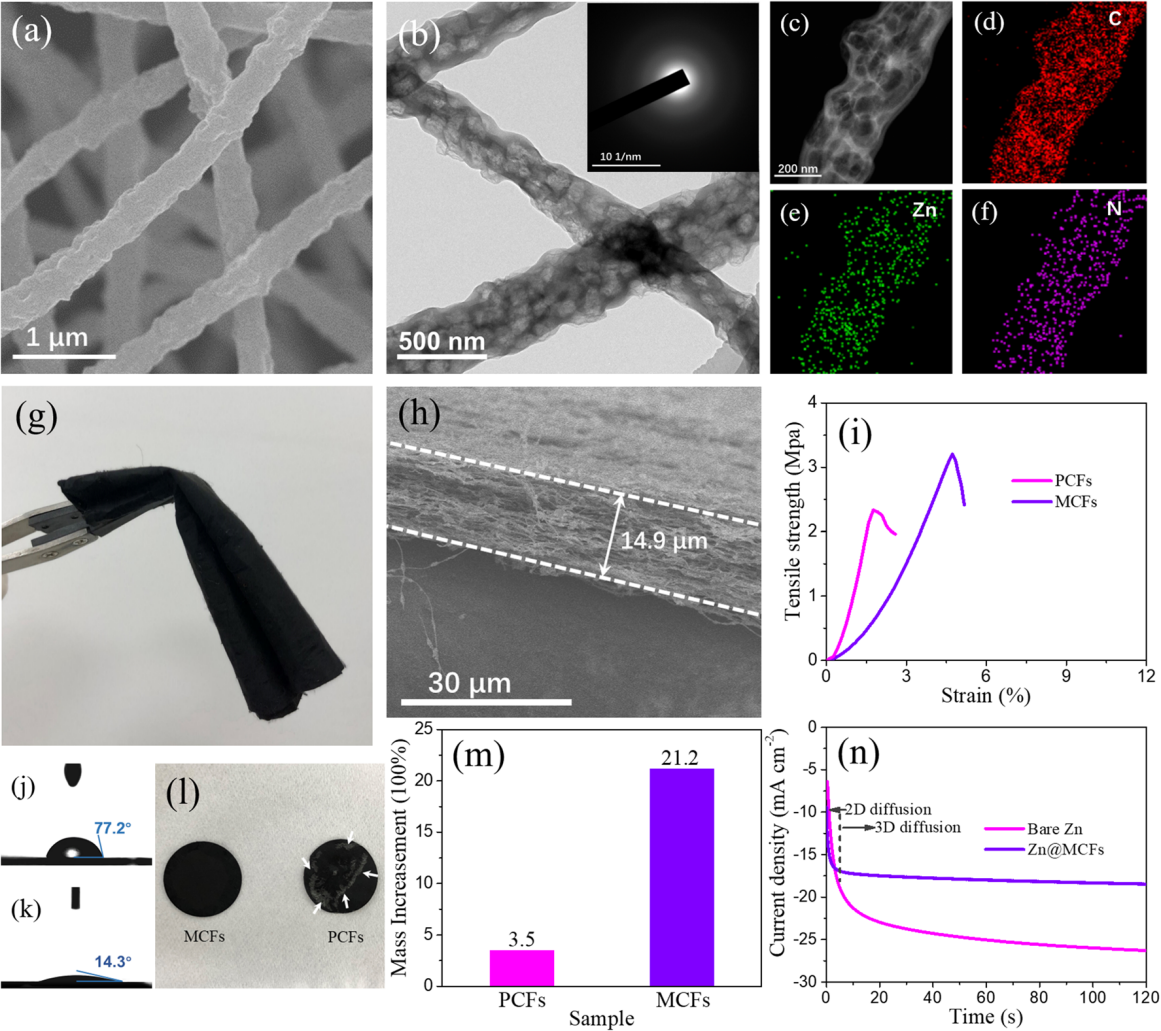
**a** SEM image of MCFs. TEM characterization result of MCFs: **b** TEM image (inset shows the SAED pattern); **c-f** HAADF image and corresponding EDS mappings of C, Zn, and N. **g** Digital photograph of MCFs cloth. **h** Side view of MCFs cloth under SEM. **i** Tensile test result of PCFs and MCFs. Contact angle test result of **j** Zn@PCFs, and **k** Zn@MCFs. **l** Digital photograph of PCFs and MCFs slices after soaked in electrolyte (2 M ZnSO_4_) for 12 h and thorough air drying. **m** Electrolyte uptake ratio of PCFs and MCFs. **n** Chronoamperometries of bare Zn and Zn@MCFs with an applied overpotential of − 120 mV

The freestanding MCFs cloth is fabricated through electrospinning technique, following by high-temperature pyrolysis. ZIF-8 particles with average particle size around 100 nm were applied as pore-forming agent and zinc source (Fig. S1). As shown in Fig. S2, the polymer cloth obtained from electrospinning shows excellent mechanical strength and flexibility. The SEM images illustrate the uniform distribution of ZIF-8 particles in the precursor fibers (Fig. S3). After pyrolysis process, ZIF-8 template collapses to form capsules, and the polymer precursor is carbonized to produce carbon nanofiber with diameter around 500 nm (Figs. 1a and S4) [35, 36]. The TEM images further reveal the detail information of morphology. Uniform hollow capsule-like structure is clearly disclosed, which supplies abundant space to immobilize the electrolyte (Figs. 1b and S5). As shown in Figs. 1c–f and S6, EDX elemental mapping illustrates the homogeneous distribution of C, Zn, O, and N elements throughout the MCFs. For comparison, PAN-derived carbon fiber (abbreviated as PCFs) cloth was prepared by the same method, except addition of ZIF-8 template. In contrast to the poriferous structure of MCFs, PCFs show a smooth surface with solid interior with uniform distribution of C, N, and O elements (Figs. S7a–e and S8). As shown in Figs. 1b, S7f and S9, XRD spectrometry and SAED (selected area electron diffraction) reveal the amorphous feature of as-synthesized carbon fibers and Zn species in MCFs [27, 37].

As Zn metal is a supportless anode, it endures dramatic volume change during plating/stripping processes. Hence, the unstable Zn-electrolyte interface probably causes the rapid exfoliation and fragmentation of interfacial layer and results in protection failure. A freestanding interface layer with good mechanical strength and flexibility can efficiently overcome this problem [38]. As shown in Figs. 1g and S10, the MCFs cloth can be folded, enwound, and bent without breakage, suggesting the good lightness and flexibility. The thickness of MCFs layer can be precisely controlled by altering the spinning time. In this work, the typical thickness of the carbonous layer is around 15 μm (Figs. 1h and S11). As displayed in Fig. 1i, the maximum tensile strength of MCFs reaches a high level of 3.2 MPa, showing obvious improvement in comparison with PCFs (2.3 MPa), which results from the mechanical enhancement effect of micropore architecture [39]. When used as interfacial layer, the freestanding MCFs layer with good mechanical strength and flexibility is supposed to overcome the problem of mechanical failure.

Moreover, the carbon network is proved to exhibit good wetting performance with small electrolyte contact angles of only 14.3° for Zn@MCFs (Fig. 1k), in contrast to 77.2° for bare Zn (Fig. 1j). The smaller contact angle of Zn@MCFs compared with Zn@PCFs is probably associated with the stronger affinity between electrolyte and Zn-contained carbon nanofibers (Figs. 1k and S12) [40]. The better wettability to electrolyte endows a smaller interfacial ion-transfer resistance, and is beneficial to the reversible Zn stripping/plating performance [41].

As confirmed by nitrogen absorption/desorption test, the MCFs exhibit a high BET area of 389.6 m^2^ g^−1^, in comparison with 299.9 m^2^ g^−1^ for PCFs (Fig. S13). The higher surface area can provide more deposition sites for Zn^2+^. More importantly, the ample inner space of MCFs significantly improves the electrolyte uptake and precludes the adverse electrolyte flow on the surface of Zn anode. The absorption amount of MCFs reaches 21.2 times of the mass after soaked in electrolyte for 12 h, in contrast to only 3.5 times for PCFs (Fig. 1m). Moreover, as shown in Fig. 1l, the electrolyte-soaked PCFs cloth separates out Zn-salt after natural drying. In contrast, although the MCFs cloth absorbs much more electrolyte, no obvious precipitate is observed on the surface, implying most of the electrolyte salt is absorbed in the capsules.

It is a broad consensus that the interface 2D ion transport is a principal reason for the selective deposition of Zn^2+^ [42]. The MCFs with high electrolyte uptake can effectually stabilize the electrolyte flow and inhibit the 2D diffusion of Zn^2+^ on the surface of electrode. To further testify the 2D diffusion inhibition effect of MCFs, we conducted chronoamperometry (CA) test (Fig. 1n). When applied an overpotential of -120 mV, the response current of bare Zn electrode keeps rising beyond 120 s, indicating a rampant and incessant 2D diffusion on the surface. The Zn^2+^ transfers freely under the drive of electrical field to selectively deposit on the lowest energy consumption sites for reduction [8]. As a result, the deposited Zn grows disorderly to from a rough and jagged surface. In contrast, the response current of Zn@MCFs electrode stables to a constant value rapidly within 5 s, implying that the detrimental 2D diffusion of Zn^2+^ is radically inhibited by the MCFs layer.

**Fig. 2:**
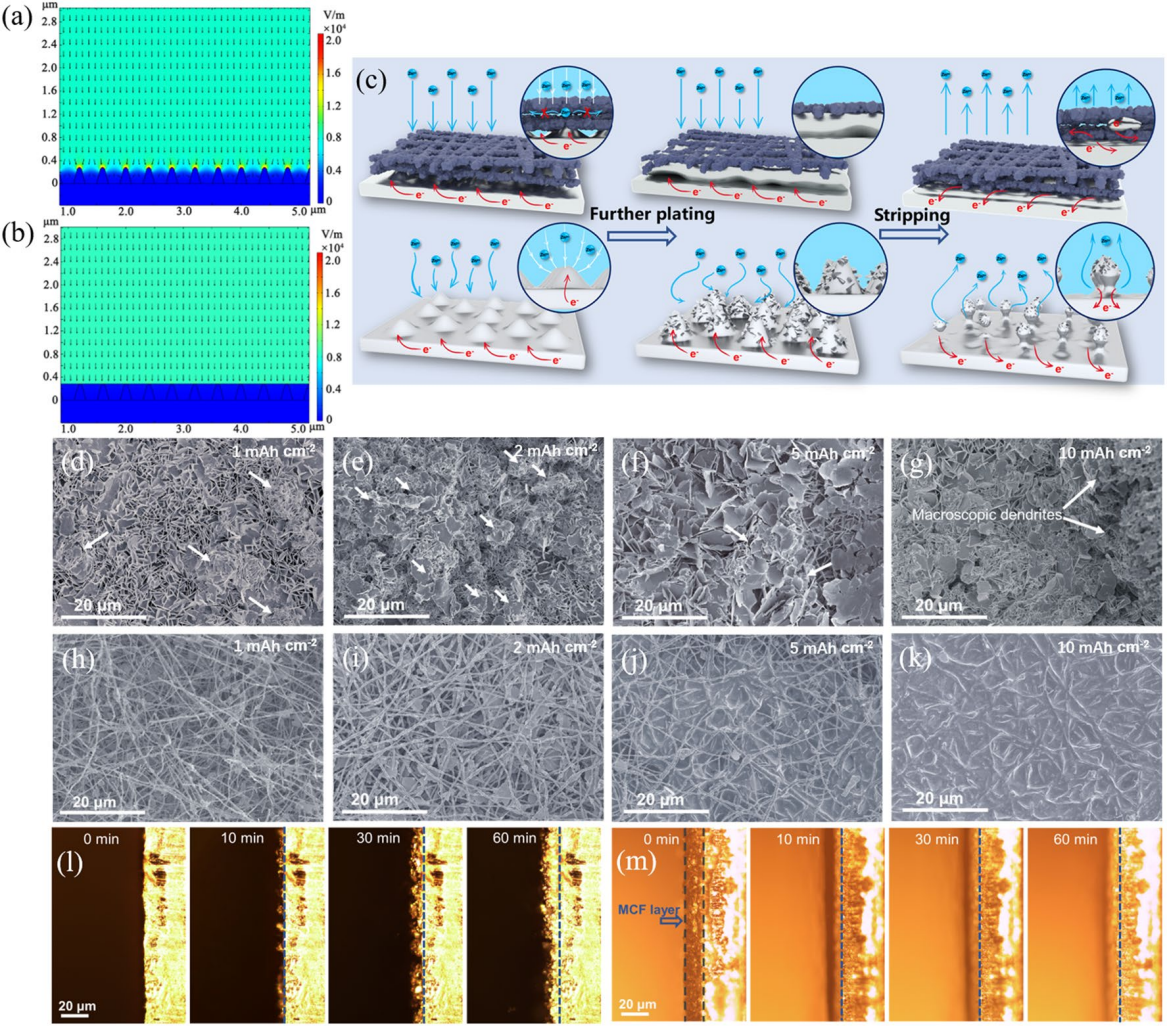
Multiphysics simulation models of the electric field distribution for **a** bare Zn and **b** Zn@MCFs. **c** Illustration of Zn plating/stripping behavior on bare Zn (lower) and Zn@MCFs (upper). Ex situ Zn deposition observation with capacities of 1, 2, 5, and 10 mAh cm^−2^ for **d–g** bare Zn, and **h–k** Zn@MCFs. In situ observation of Zn deposition at 5 mA cm^−2^ after 0, 10, 30, and 60 min for **l** bare Zn, and **m** Zn@MCFs

In order to evaluate the effectiveness of MCFs in adjusting Zn deposition and protecting Zn anode, we tested the electrochemical behaviors of bare Zn foil, Zn@PCFs, and Zn@MCFs in symmetric batteries. As shown in Fig. S14, the Zn foils used in this work show a flat surface with slight scratches resulting from polishing. Consistent with previous reports, SEM images of surface deposition morphology depict that the bare Zn foil suffers severe dendrite growth during plating (Fig. 2d–g) [43]. As marked by arrows, the vertically grown zinc sheets can easily induce uneven deposition of zinc to form dendrites (Fig. 2d). With plating going on, the dendrites grow up rapidly and evolve into macroscopic branched crystals after a deposition capacity of 10 mAh cm^−2^ (Fig. 2e–g). In sharp contrast, MCFs can regulate the homogeneous and dense deposition of Zn^2+^ from the bottom up, thereby eliminating Zn dendrites (Fig. 2h–k). Even under a high deposition of 10 mAh cm^−2^, the newly deposited Zn layer still maintains a flat surface without any sign of dendrite growth (Fig. 2k). As investigated by XRD, despite a more negative binding energy of Zn(101), the deposited Zn on Zn@MCFs anodes exhibit a preferential deposition of plane (002), which is induced by the low mismatch between Zn (002) plane and the carbon substrate (Fig. S15) [44, 45]. The TEM test further reveals that the deposited Zn on Zn@MCFs consists of microcrystals to form densely stacked lamellar structure. SAED and HRTEM further reveal the dominant exposed (002) crystal plane (Fig. S16). The parallel growing (002) facet is propitious to form dense-packed plating layer, thereby reducing the potential risk of dendrite growth [43]. For Zn@PCFs anode, although the dendrite growth is inhibited in comparison to bare Zn, it fails to avoid formation of adverse crystal protrusions, which may further evolve into dendrites as the cycle continues (Fig. S17).

The in situ optical microscope visualization supplies a more intuitive observation of Zn plating behavior on bare Zn and Zn@MCFs. As shown in Fig. 2l, irregular nucleation appears on the bare Zn in merely 10 min, and gradually develops into a rough surface covered by dendrites. In contrast, the Zn@MCFs anode exhibits bottom-up deposition and maintains a smooth surface throughout the plating process (Fig. 2m). In addition, the thickness of newly deposited Zn layer on Zn@MCFs is much lower than that on bare Zn, suggesting a denser Zn deposition on Zn@MCFs anode. More intuitive observation of deposition behavior can be presented by videos (180 times faster). Again, the bare Zn endures rampant dendrite growth during plating (Video S1), while the Zn@MCFs anode shows flat and bottom-up Zn plating (Video S2).

A high-quality plating layer requires low nucleation overpotential and uniform Zn^2+^ flux on the Zn-electrolyte interface [5, 46]. As illustrations depict in Fig. 2c, the electrically conductive MCFs layer with high specific surface area supplies ample nucleation sites, endowing a lower overpotential. More importantly, the MCFs layer efficiently homogenize the Zn^2+^ flux by simultaneously regulating interface electric field and inhibiting 2D diffusion of electrolyte flow. To further clarify the role of MCFs layer in adjusting interfacial electric field and Zn^2+^ distribution, we carried out a multiphysics theoretical simulation. As displayed in Fig. 2b, simulation result indicates the uniform electric field on the surface of Zn@MCFs anode, which guides to form uniform Zn^2+^ flux distribution (Fig. S18b). In contrast, protuberances result from uneven nucleation on bare Zn surface enhance the local electric field on the tips and generate an intensity gradient (Fig. 2a). Such locally enhanced electric field further impels more Zn^2+^ to the tips, thereby accelerating the formation and growth of sharp dendrites (Figs. 2c and S18a) [47]. In the stripping process, Zn metal in the root of dendrite trends to have a prior donation of electron, which leads to dendrite fracture at root and formation of “dead Zn”. The “dead Zn” significantly reduces the coulombic efficiency, also improves risk of short circuit [48], whereas the conductive MCFs skeleton supplies robust electrical contact for the revival of possibly generated “dead Zn”, which can be demonstrated by in situ stripping observation (Figs. 2c and S19). Therefore, the MCFs layer is also promised to remarkably improve the coulombic efficiency of Zn anode.

**Fig. 3:**
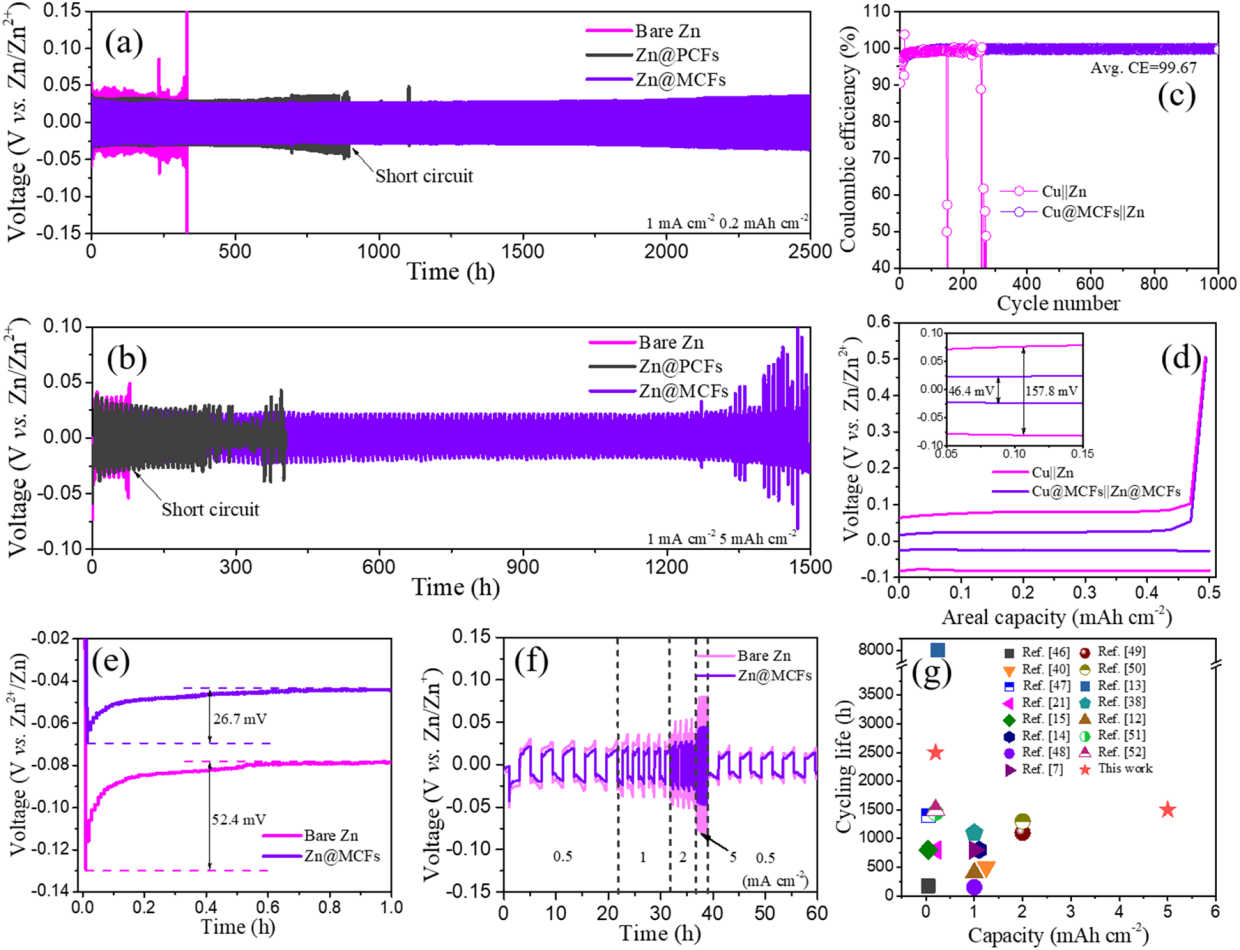
Galvanostatic plating/stripping test of bare Zn, Zn@PCFs and Zn@MCFs in symmetric cells with a capacity of **a** 0.2 mAh cm^−2^ at 1 mA cm^−2^, **b** 5 mAh cm^−2^ at 1 mA cm^−2^. **c** Coulombic efficiency of Zn plating/stripping in Cu||Zn asymmetrical cells with/without protection of MCFs. **d** Voltage–capacity curves of Cu||Zn and Cu@MCFs||Zn@MCFs asymmetrical cells at 50th cycle. **e** Voltage–time curves of Zn plating on bare Zn and Zn@MCFs at 5 mA cm^−2^. **f** Rate performance of bare Zn and Zn@MCFs symmetric cells. **g** Comparison of plating/stripping performance of Zn@MCFs anode with some previously reported anodes modified by various interfacial layers

To assess the practical effect of MCFs layer in regulating the Zn plating behavior, we assembled and tested the symmetric cells. Figure 3a–b shows the galvanostatic voltage profiles of bare Zn, Zn@PCFs, and Zn@MCFs symmetric cells with Zn plating capacities of 0.2 and 5 mAh cm^−2^, respectively. Remarkably, the Zn@MCFs||Zn@MCFs cell exhibits steady voltage profiles with a small hysteresis beyond 2500 h at 1 mA cm^−2^. However, the bare Zn||bare Zn cell holds only 332 h before collapse, while the Zn@PCFs symmetric cell delivers a lifespan of ~ 903 h (Fig. 3a). In addition, the voltage profile of Zn@MCFs||Zn@MCFs cell is much smoother than that of bare Zn and Zn@PCFs, implying a more stable interface. The advantages of MCFs interfacial layer can be further amplified under a higher plating capacity of 5 mAh cm^−2^. As displayed in Fig. 3b, the bare Zn cell collapses after only 75 h, as a result of dendrite-triggered internal short-circuit. Meanwhile, the operation time of Zn@PCFs cell also drops dramatically to 264 h, only 29% retain of the lifespan under 0.2 mAh cm^−2^. In contrast, the Zn@MCFs cell woks steadily up to 1500 h. The fluctuation of overpotential in the late cycles probably results from the instability of the electrode–electrolyte interface caused by aerogenesis (Fig. S20). Of note, a lifespan of 190 h is achieved under an ultrahigh plating capacity of 10 mAh cm^−2^ at 10 mA cm^−2^ (Fig. S21). As intuitive contrast shown in Fig. 3g, Zn@MCFs anode performs much better in contrast to recently reported zinc anodes modified by various interfacial layers (Table S1) [8, 13–16, 22, 40, 42, 49–55].

The coulombic efficiency (CE) is another vital parameter to assess the reversibility of electrode. CE test was carried out in asymmetrical cell using Cu foil as counter electrode with a plating capacity of 0.5 mAh cm^−2^ at 2 mA cm^−2^. As shown in Fig. 3c, the MCFs-decorated half cell can deliver an ultrahigh average coulombic efficiency of 99.67% up to 1000 cycles. Whereas, the CE of Cu||Zn half cell degenerates after only 260 cycles, which results from the continuous consumption of Zn source by side reactions and formation of “dead Zn”. Furthermore, as displayed in Fig. 3d, Cu@MCFs||Zn asymmetrical cell exhibits a remarkably low voltage hysteresis of 46.4 mV. In contrast, Cu||Zn cell shows a poor plating kinetics reflected by the high voltage hysteresis of 157.8 mV. The plating/stripping kinetics optimization effect of MCFs layer is further demonstrated by the reduced nucleation and platform overpotentials in Zn@MCFs symmetric battery. As shown in Fig. 3e, Zn@MCFs symmetric cell displays a nucleation overpotential of only 26.7 mV at 5 mA cm^−2^, much lower than that of bare Zn symmetric cell (52.4 mV). Moreover, the rate test reveals that Zn@MCFs symmetric cell shows much lower voltage hysteresis than bare Zn cell (Figs. 3f and S22). In summary, by revival of “dead Zn” and reduction of overpotential, the MCFs have been verified as an effective modification layer to improve electrochemical reversibility and kinetics performance.

**Fig. 4:**
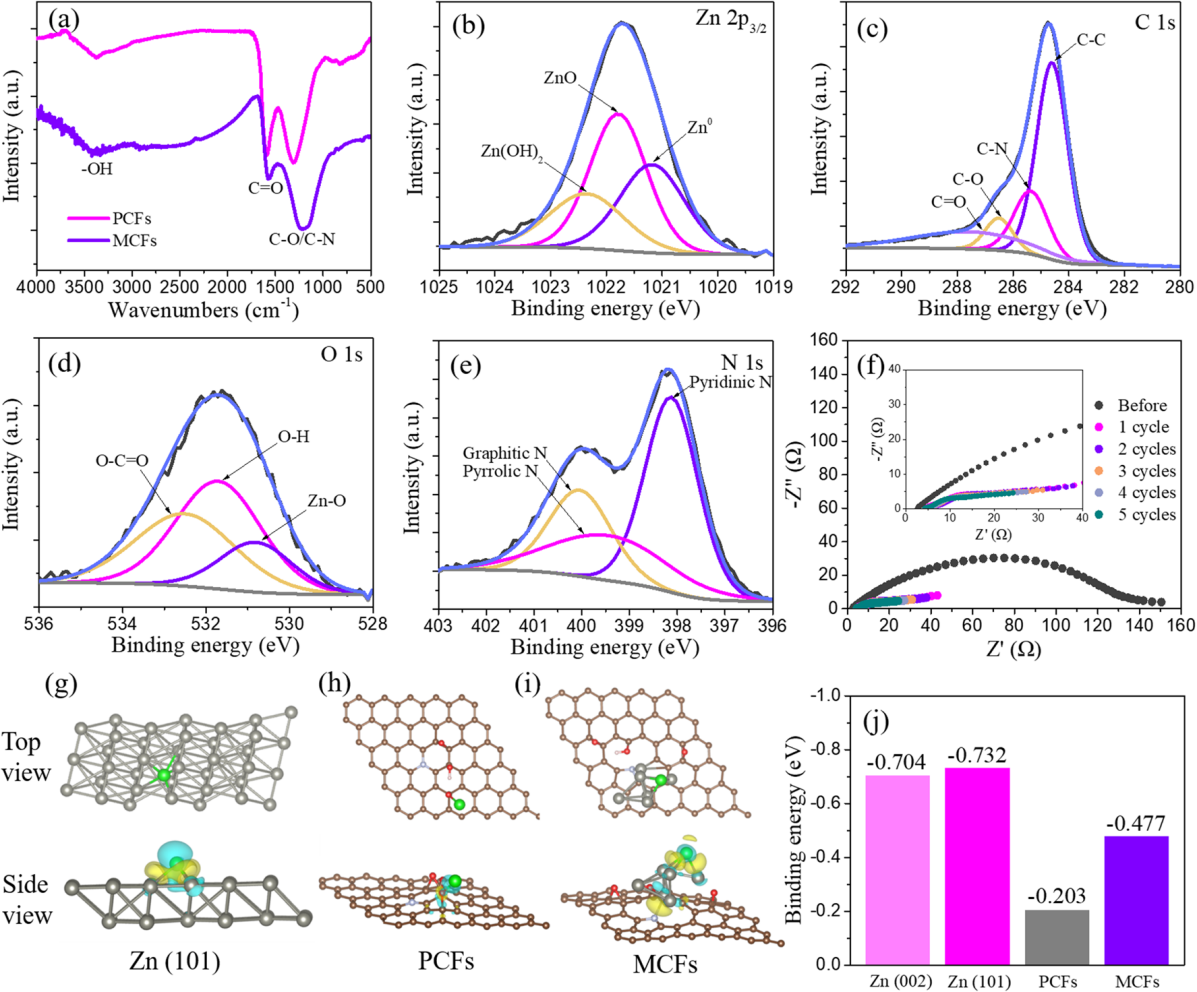
**a** FTIR spectra of PCFs and MCFs. **b–e** XPS spectra of Zn 2*p*_3/2_, C 1*s*, O 1*s* and N 1*s* for MCFs, respectively. **f** Electrochemical impedances spectra of Zn@MCFs symmetrical cell before and after 1, 2, 3, 4, 5 cycles. **g–i** Surface atomic configuration models of zinc onto Zn (101), PCFs and MCFs and corresponding electron cloud distributions, respectively. **j** The calculated binding energy comparison of Zn onto different substrates

For a more penetrating comprehension of the Zn deposition behavior on various anodes, we performed DFT calculations based on first principles. First, both FTIR and XPS tests were carried out to reveal the surface chemical compositions on PCFs and MCFs. Coincident with the HAADF-EDS result, the XPS demonstrate the existence of zinc in MCFs and nitrogen both in PCFs and MCFs (Fig. S23). As depicted in Fig. 4a, FTIR spectra show three main peaks located around 1307/1212, 1592/1565, and 3370/3380 cm^−1^ for PCFs and MCFs, respectively, which can be attributed to C-O/C-N, C = O, and -OH stretching vibrations [24, 56]. The red shift of FTIR peaks in MCFs results from the interactions between zinc clusters and surface functional groups [57]. More detailed chemical information can be collected from the magnified XPS spectra. As shown in Fig. 4b, the Zn 2*p* spectrum can be deconvoluted into three peaks, conforming three components: Zn^0^ at 1021.2 eV, ZnO at 1021.8 eV, and Zn(OH)_2_ at 1022.4 eV [27]. The C 1*s* and O 1*s* spectra further confirm the oxygen-containing functional groups on carbon substrates, including C-O, C = O, O-C = O, and O–H, consistent with the FTIR result (Figs. 4c–d and S24a) [24, 58–60]. As for N 1*s* spectra, three component peaks can be matched corresponding to pyridinic N (398.1 eV), pyrrolic N (399.4 eV), and graphitic N (400.1 eV), respectively (Figs. 4e and S24b) [61–63].

In order to compare the binding energies of Zn onto different substrates, we constructed the preferred surface atomic configuration of PCFs and MCFs according to FTIR and XPS results (Fig. 4h–i). For bare Zn substrate, preferentially oriented (101) and (002) crystal planes are chosen according to the XRD results (Figs. 4g, S25 and S26). For fair comparison, three sites are calculated to identify the preferred configuration of Zn clusters on MCFs (Fig. S27). The binding energy values represent the change of total energy after Zn deposition, of which a more negative value indicates easier deposition. As depicted in Fig. 4j, the Zn (101) and Zn (002) planes show the most negative binding energies among three substrates. In contrast, PCFs exhibit a binding energy value of -0.203 eV, while the MCFs show moderate value of -0.477 eV. In addition, local differential charge densities further indicate the week affiliation between Zn atom and PCFs, coincident with previously reported carbonaceous substrates [64–66]. The introduction of Zn clusters on MCFs can effectively enhance the electronic interaction between Zn atom with the substrate (Figs. 4g–i and S28). The DFT calculation reveals the moderate zincophilicity of MCFs, enabling a relatively low nucleation barrier and boosting the uniform bottom-up Zn deposition. Moreover, the low interfacial impedance value suggests the excellent electrical contact between MCFs layer and Zn plate. As shown in Figs. 4f and S29, the interfacial impedance decreases remarkably from 457.3 Ω in bare Zn symmetrical cell to 129.3 Ω in Zn@MCFs symmetrical cell. In addition, the interfacial impedance of Zn@MCFs cell significantly reduces to 26.6 Ω after initial plating/stripping process, and further decreases to 14.9 Ω after 5 cycles. In contrast, the interfacial impedance in bare Zn symmetrical cell increases gradually from 37.2 Ω after initial cycle to 46.4 Ω after 5 cycles, which probably results from the generation of insulating by-products during cycling. The side view of deposited Zn@MCFs anode also shows the tight adhesion of MCFs layer on Zn foil, and the deposited Zn works as “binder” to contact the MCFs layer and the Zn foil (Fig. S30). These results indicate the good electrical contact reliability between MCFs layer and Zn foil.

For electrochemical performance test, lab-prepared MnO_2_ and commercial MnO_2_ (99%, Aladdin) were employed as cathodes and paired with above-mentioned anodes to assemble aqueous zinc ion batteries. As detected by XRD, the MnO_2_ synthesized according to previous report belongs to α-phase, and exhibits a novel needlelike morphology (Fig. S31) [28]. The large length-diameter ratio enables the rapid Zn^2+^ diffusion and charge transfer, promising an enhanced electrochemical performance.

**Fig. 5:**
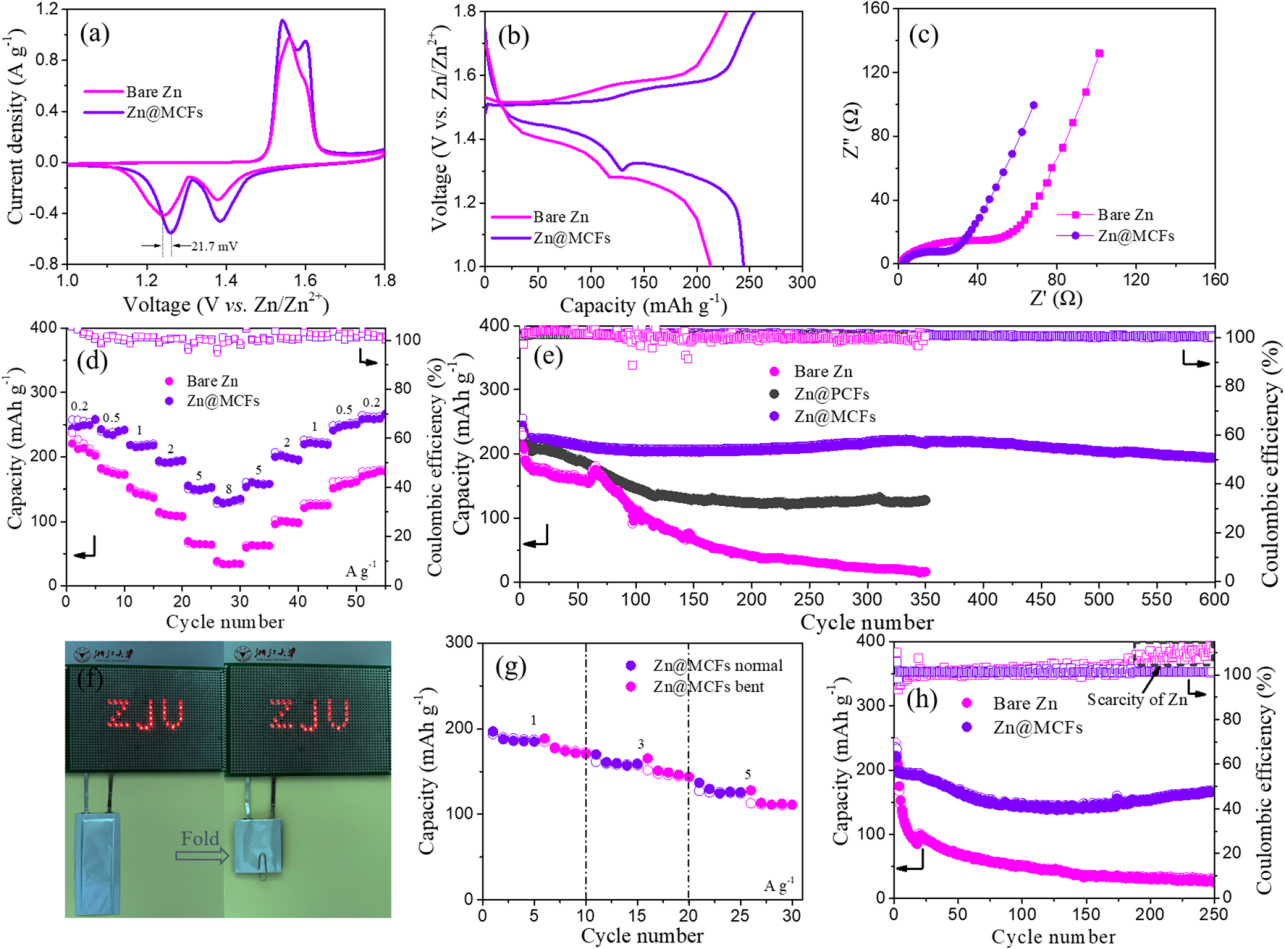
Electrochemical performance of aqueous Zn||α-MnO_2_ batteries. **a** CV curves of bare Zn||α-MnO_2_ and Zn@MCFs||α-MnO_2_ at 0.1 mV s^−1^. **b** Voltage–capacity curves at 0.2 A g^−1^. **c** EIS spectra before cycling. **d** Rate performance ranging from 0.2 to 8 A g^−1^. **e** Long-span cycling performance of Zn||MnO_2_ batteries using various anodes (activating at 0.2 A g^−1^ for two cycles and cycling at 1 A g^−1^). **f** Optical photographs of 30 red LEDs lit by Zn@MCFs||α-MnO_2_ pouch batteries under normal and bent (180°) states. **g** Rate performance of Zn@MCFs||α-MnO_2_ pouch batteries under normal and bent states. **h** Cycling performance of Zn||α-MnO_2_ and Zn@MCFs||α-MnO_2_ pouch batteries at 1 A g^−1^ after activation at 0.2 A g^−1^ for two cycles

As shown in Fig. 5a, CV curves present typical redox peaks corresponding to Zn||α-MnO_2_ battery. Two pairs of distinct peaks reflect the successive two-step reaction with H^+^ and Zn^2+^ coinsertion [67]. The Zn@MCFs||α-MnO_2_ battery shows a lower overpotential than Zn||α-MnO_2_ cell. Consistent with the CV curves, there are two plateaus observed in the voltage–capacity curves. The voltage–capacity curves of Zn@MCFs||α-MnO_2_ battery also show significantly reduced voltage hysteresis in comparison with Zn||α-MnO_2_ battery (Fig. 5b). As shown in Fig. 5d, the capacity of Zn@MCFs||α-MnO_2_ battery drops tardily as the employed current density increases, and it still delivers a high capacity of 132 mAh g^−1^ at an ultrahigh rate of 8 A g^−1^. With the current density falling back gradually, the capacity fully recovers to form a perfect v-shape, indicating the superior interfacial stability and kinetics properties of Zn@MCFs anode. In contrast, the capacity of Zn||α-MnO_2_ battery declines rapidly as the current density increases, only maintaining 33.9 mAh g^−1^ at 8 A g^−1^. The enhanced rate performance can be attributed to the reduced charge transfer impedance after decorating MCFs interfacial layer (Fig. 5c).

Besides superior rate performance, the Zn@MCFs||α-MnO_2_ battery also delivers excellent long-span cycling stability (Fig. 5e). The MCFs layer stabilizes interfacial electric field and Zn^2+^ flow, as well reduces overpotentials, therefore effectually inhibiting Zn dendrite and side reactions. As a result, the Zn@MCFs||α-MnO_2_ battery delivers a high initial capacity of 236.1 mAh g^−1^ at 1 A g^−1^ and retains 195.5 mAh g^−1^ after 600 cycles, with a retention rate of 82.8%. However, the Zn@PCFs||α-MnO_2_ and Zn||α-MnO_2_ batteries drops rapidly to 127.6 and 21.9 mAh g^−1^, respectively, after 300 cycles. The high and immobile coulombic efficiency further testifies the interfacial stability of Zn@MCFs anode. Besides, we also matched the bare Zn and Zn@MCFs with commercial MnO_2_ (98%, Aladdin) to assemble aqueous full batteries. The commercial MnO_2_ exhibits an irregular morphology of micron scale particles (Fig. S32). As revealed in Fig. S33b, the batteries paired with commercial MnO_2_ exhibit long activization processes, which can be ascribed to the sluggish kinetics of commercial micron-sized MnO_2_. According to the voltage–capacity profiles, CV curves and EIS results (Fig. S34), the kinetics superiority of Zn@MCFs anode can be further amplified in commercial cathode materials with poor kinetics properties. To demonstrate the mechanical stability of MCFs layer in practical application, we characterized the cycled MCFs. As depicted in Fig. S35, MCFs layer maintains intact network structure after long-span cycles in symmetric and Zn||α-MnO_2_ batteries. Moreover, as detected by TEM, the MCFs keep multi-hole structure after long-span test in Zn||α-MnO_2_ battery (Fig. S36a). The residual Zn nanocrystals in the nanofibers prove that a part of zinc is deposited within the pores, which enables dense and dendrite-free plating (Fig. S36b-c).

More importantly, for practicality evaluation, we assembled pouch batteries and tested with controlled depth of discharge (DOD). Excessive Zn will cover up the negative effects of side reactions and dendrite growth, as well severely reduce the energy density. In order to objectively evaluate the performance, the pouch batteries were assembled under Zn-scarce state by using 10 μm Zn foil (6.95 mg cm^−2^, 5.7 mAh cm^−2^). By adjusting the loading mass of cathode, the DOD is controlled around 33%. As-assembled Zn@MCFs||α-MnO_2_ pouch batteries can easily light up 30 LEDs under normal and bent (90° and 180°) states (Figs. 5f and S37c). In addition, only slight capacity decline is observed under bent state (180°) in contrast to normalcy at various current densities, suggesting the excellent mechanical flexibility and electrochemical stability. The 2 × 3 cm^2^ pouch battery maintains a considerable capacity of 125.5 mAh g^−1^ at 5 A g^−1^, equivalent to 83% of the value in coin battery (Figs. 5g and S38a). As depicted in Fig. 5h, Zn@MCFs||α-MnO_2_ pouch battery delivers an initial discharge capacity around 199.8 mAh g^−1^, corresponding to an energy density of 154.3 Wh kg^−1^ (based on the mass of cathode and anode). Moreover, it gives excellent cycling performance, maintaining 165.7 mAh g^−1^ (129.6 Wh kg^−1^) after 250 cycles. The capacity uplift after 150 cycles may come from the gradual activation of cathode materials (Figs. 5h and S38b). In contrast, although the Zn||α-MnO_2_ pouch battery gives out similar initial capacity value, it drops rapidly to only 26.8 mAh g^−1^ after 250 cycles, which results from the quick depletion of limited Zn source [66]. The pouch battery test further demonstrates the practicability of Zn@MCFs anodes.

## Conclusion

In summary, a freestanding interfacial layer composed of multicapsular carbon fibers is innovatively proposed to protect the Zn anode in neutral aqueous zinc ion batteries. The electron conductive MCFs layer with high electrolyte uptake effectively homogenizes the interface electric field and inhibits the 2D diffusion of Zn^2+^ flow, thereby endowing the anode with low overpotentials and uniform Zn deposition. Furthermore, the in/ex situ plating observation and theoretical calculation verify that the moderate affinity between MCFs and Zn^2+^ leads to the bottom-up and homogeneous Zn deposition on the Zn/MCFs interface, therefore realizing high-capacity plating. As a result, the MCFs layer protected Zn anode exhibits distinct advantages including ultralong cycling life up to 1500 h under 5 mAh cm^−2^ in symmetric battery, high coulombic efficiency of 99.7% in asymmetrical cell, and superior cycling stability with 82.8% capacity retention after 600 cycles in Zn@MCFs||α-MnO_2_ battery. More remarkably, the Zn@MCFs||α-MnO_2_ pouch battery delivers 154.3 Wh kg^−1^ and cycles stably over 250 cycles. Distinctly, we have demonstrated the feasibility of MCFs in the modification of Zn anode in aqueous zinc ion battery, and we believe this work will initiate the research of freestanding interfacial layer in modification of metal-based anodes.

## Supplementary Information

Below is the link to the electronic supplementary material.

Supplementary file1 (PDF 2355 kb)

Supplementary file2 (MP4 19823 kb)

Supplementary file3 (MP4 21028 kb)
