# Supplementary material for: Freestanding and Flexible Interfacial Layer Enables Bottom-Up Zn Deposition Toward Dendrite-Free Aqueous Zn-Ion Batteries
Source: Nanomicro Lett. 2022 Sep 1;14:180. doi: 10.1007/s40820-022-00921-6 (PMC9437200; doi:10.1007/s40820-022-00921-6)
Supplement: Supplementary file 1 — Supplementary file1 (PDF 2355 kb) [file 40820_2022_921_MOESM1_ESM.pdf]

Supporting Information for

Freestanding and Flexible Interfacial Layer Enables Bottom-Up Zn Deposition Towards Dendrite-Free Aqueous Zn-Ion Batteries

Hangjun Ying[1,] *, Pengfei Huang[1], Zhao Zhang[1], Shunlong Zhang[1], Qizhen Han[1], Zhihao Zhang[1], Jianli Wang[1], Wei-Qiang Han[1,] *

[1]School of Materials Science and Engineering, Zhejiang University, Hangzhou 310027, P. R. China

*Corresponding authors. E-mail: yinghangjun@zju.edu.cn (Hangjun Ying), hanwq@zju.edu.cn (Wei-Qiang Han)

Supplementary Figures and Table

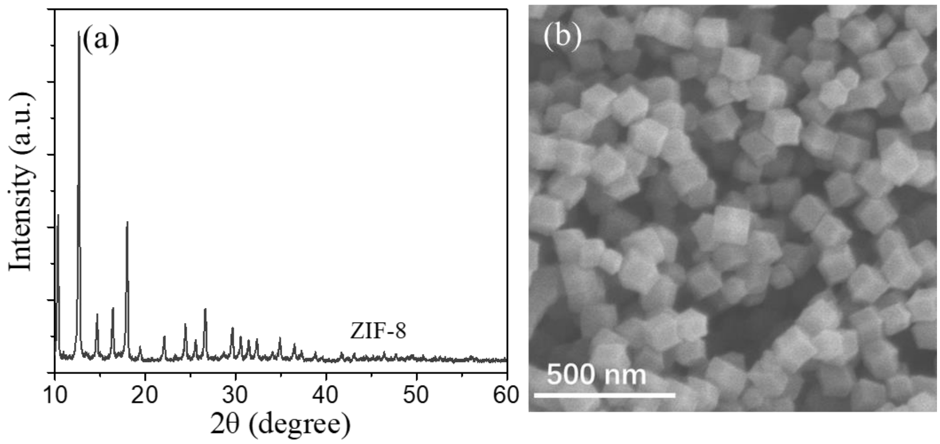

Fig. S1 (**a**) XRD pattern and (**b**) SEM image of ZIF-8 template

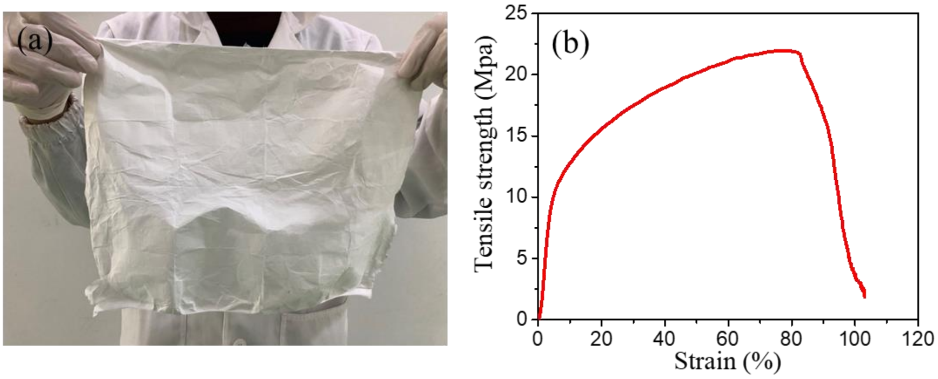

Fig. S2 (**a**) Digital photograph and (**b**) tensile test result of electrospun precursor cloth

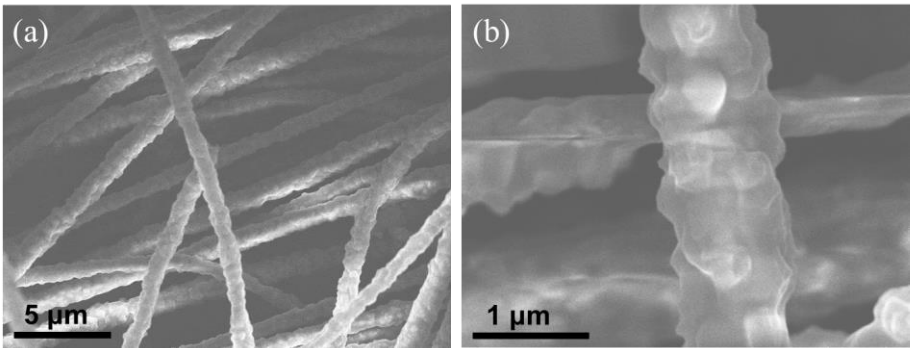

Fig. S3 SEM images at different magnifications of ZIF-8@PAN precursor nanofibers

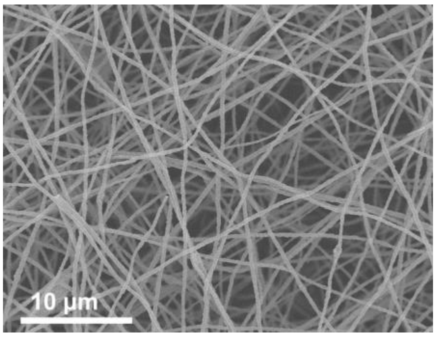

Fig. S4 SEM image of MCFs

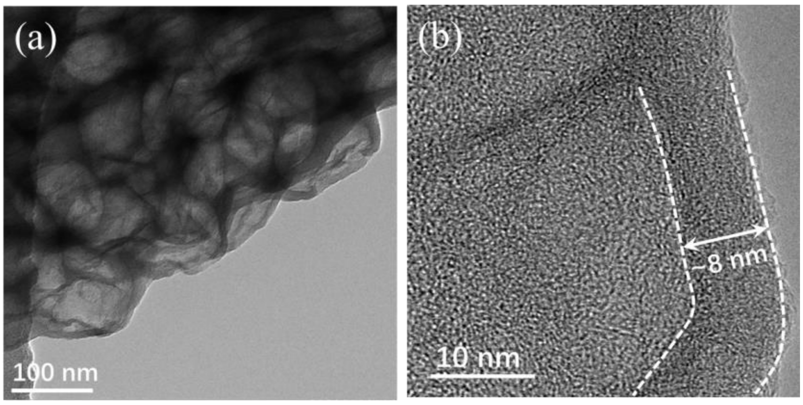

Fig. S5 (**a**) TEM and (**b**) HRTEM images of MCFs

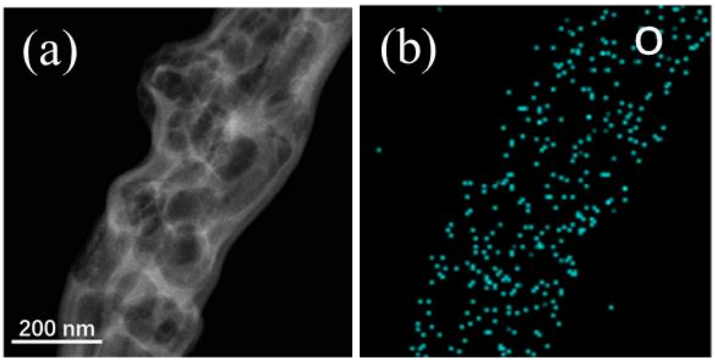

Fig. S6 (**a**) HAADF image of MCFs and (**b**) corresponding EDS mapping of O

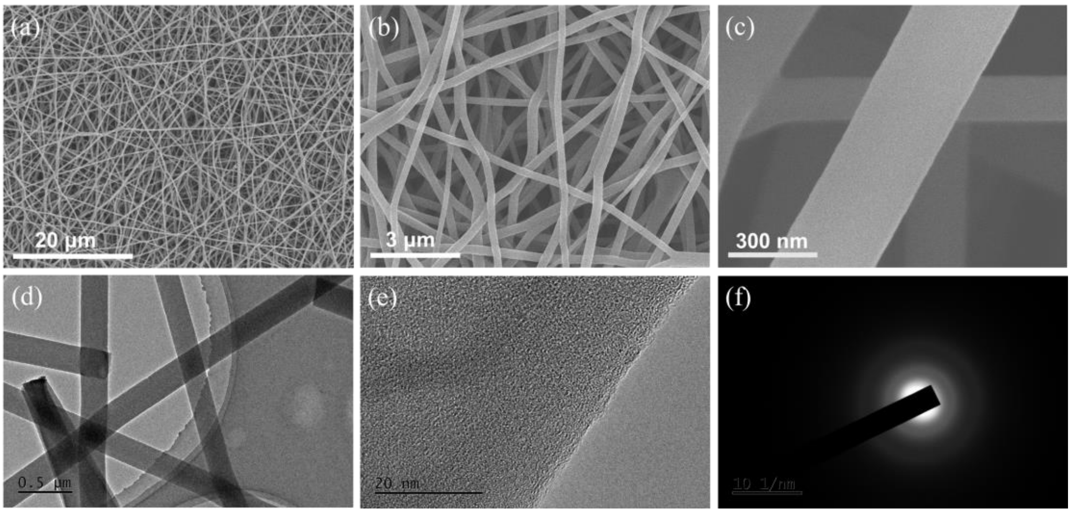

Fig. S7 (**a-c**) SEM images of PCFs at different magnifications. (**d-f**) TEM, HRTEM, and SAED images of PCFs

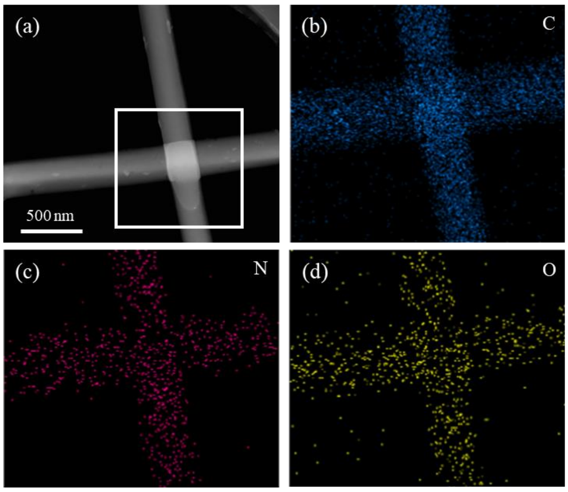

Fig. S8 HAADF image of PCFs and corresponding EDS mappings of C, N and O

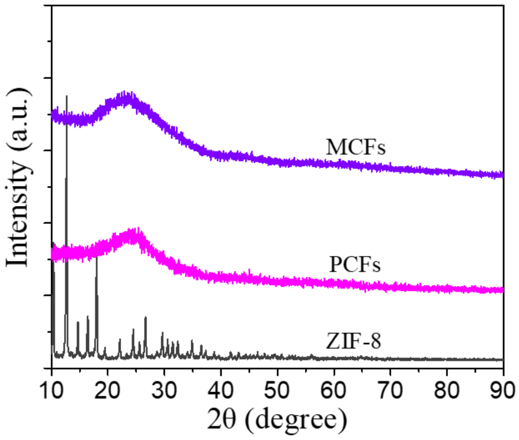

Fig. S9 XRD patterns of ZIF-8, PCFs and MCFs

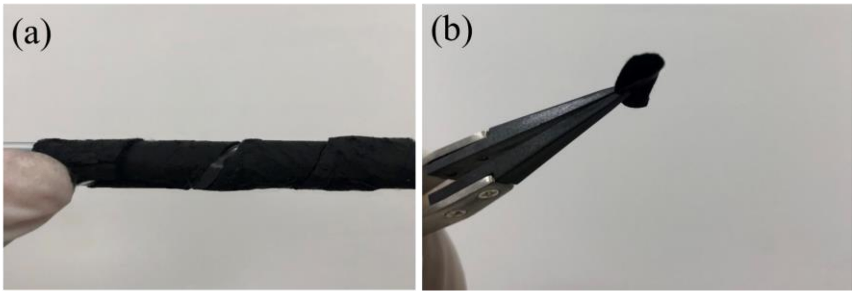

Fig. S10 Digital photographs of MCFs layer under (**a**) enwound, and (**b**) bent states

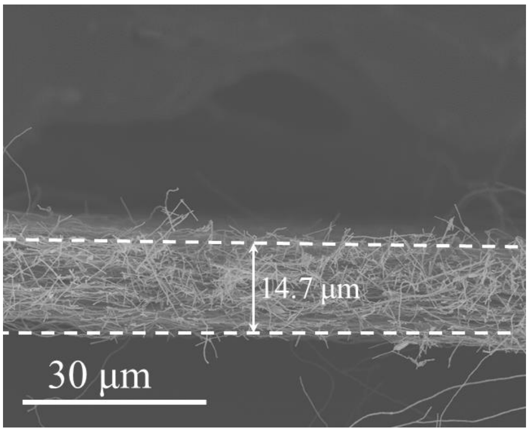

Fig. S11 Side view of PCFs cloth under SEM

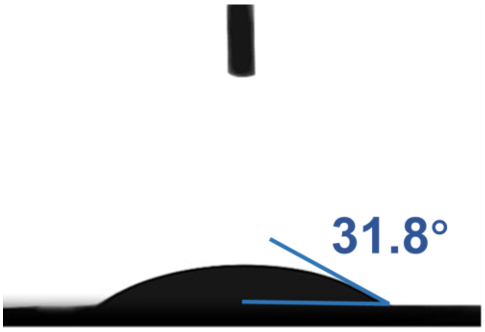

Fig. S12 Instantaneous contact angle of electrolyte (2M $ZnSO_4$) on the surface of Zn@PCFs anode

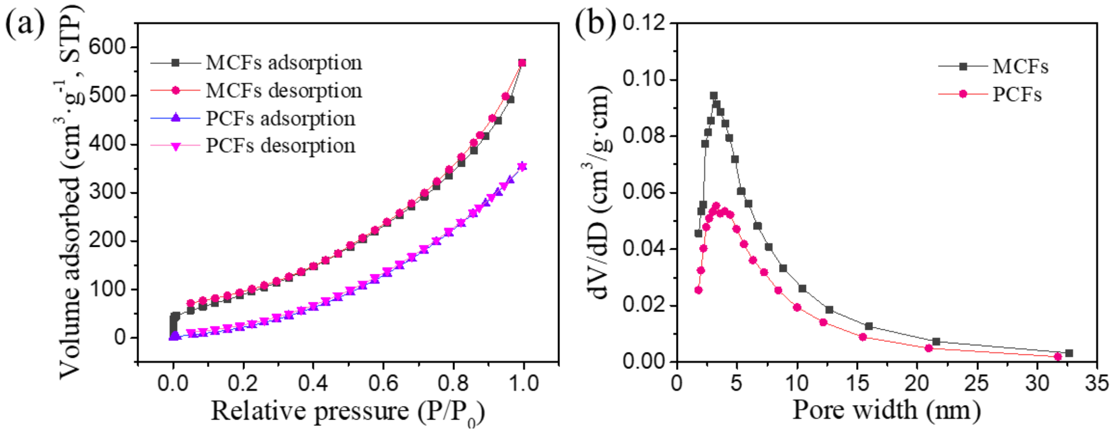

Fig. S13 (**a**) Isothermal nitrogen adsorption/desorption curves, and (**b**) aperture distribution curves of MCFs and PCFs

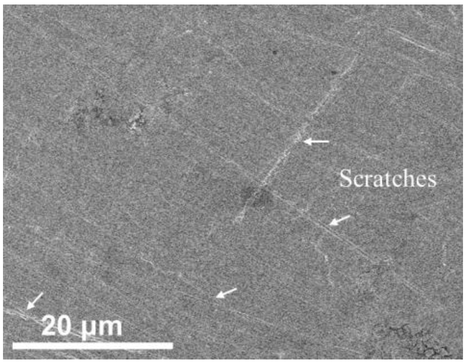

Fig. S14 SEM image of the bare Zn surface before cycling

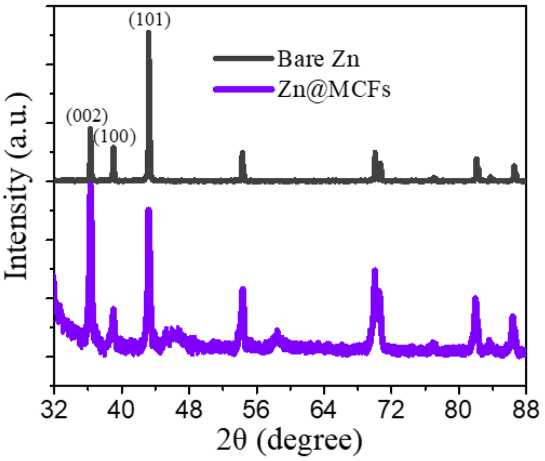

Fig. S15 XRD patterns of deposited Zn on bare Zn and Zn@MCFs anodes (5 mA cm^{-2}, 1 h)

In contrast to bare Zn, the relative peak intensity of plane (101) of deposited Zn on MCFs decreases, while plane (002) enhances dramatically, suggesting a preferential deposition of plane (002) on Zn@MCFs anode. The relative texture coefficients (RTCs) of can be calculated using the following formula [S1]:

$$RCT_{(hkl)}=\frac{I_{(hkl)}/I_{0(hkl)}}{\Sigma(I_{(hkl)}/I_{0(hkl)})}\times 100$$

Where $I_{(hkl)}$ is the intensity of plane (hkl) obtained from the samples, and $I_{0(hkl)}$ is the intensity of plane (hkl) from the standard PDF cards. Accordingly, $RCT_{(002)}$ increases from 8.02 of bare Zn to 12.45 of Zn@MCFs, while $RCT_{(101)}$ decreases from 12.09 of bare Zn to 5.59 of Zn@MCFs, indicating more (002) plane is exposed in Zn@MCFs. Despite Zn(101) shows more negative binding energy, the tendency of preferential deposition of Zn (002) plane can be attributed to the low lattice mismatch between Zn(002) and the carbon matrix, which can induce the preferential growth of Zn(002) plane. The regulation of crystal plane orientation of deposited Zn is also observed in previous reported carbonous modification layers or substrates [S2-S5].

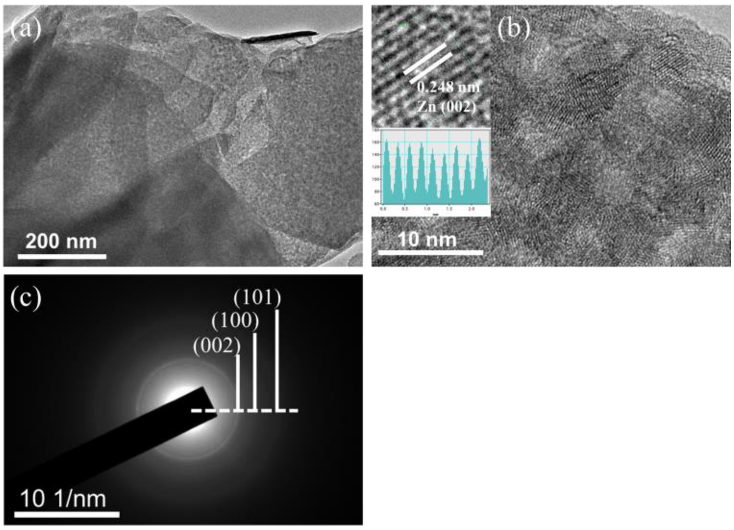

Fig. S16 (**a**) TEM image, (**b**) HRTEM image (insets show the magnified lattice fringes and plane spacing measurement result), and (**c**) SAED pattern of deposited Zn crystals on Zn@MCFs

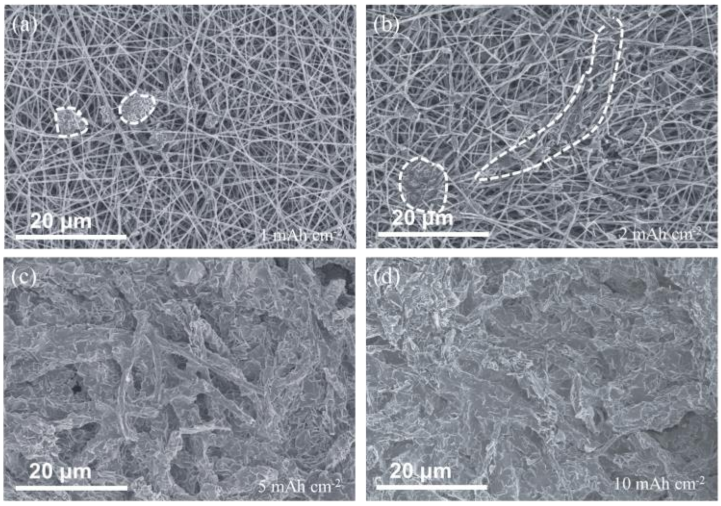

Fig. S17 The deposition morphology of Zn on Zn@PCFs anode with capacities of (**a**) 1, (**b**) 2, (**c**) 5, and (**d**) 10 mAh cm^{-2}

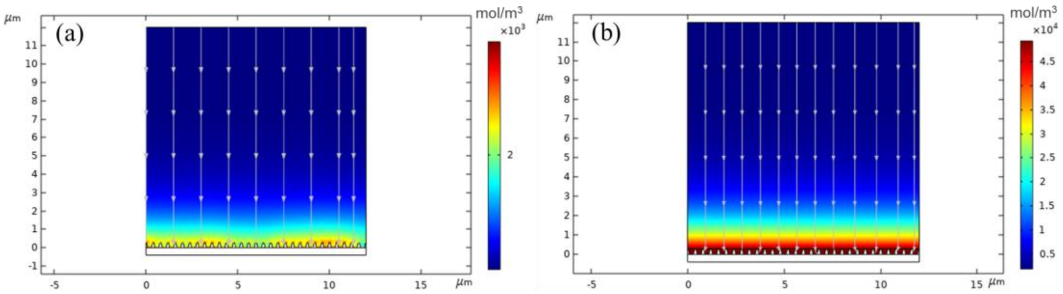

Fig. S18 Simulation results of Zn^{2+} concentration distribution on the surface of (**a**) bare Zn, and (**b**) Zn@MCFs

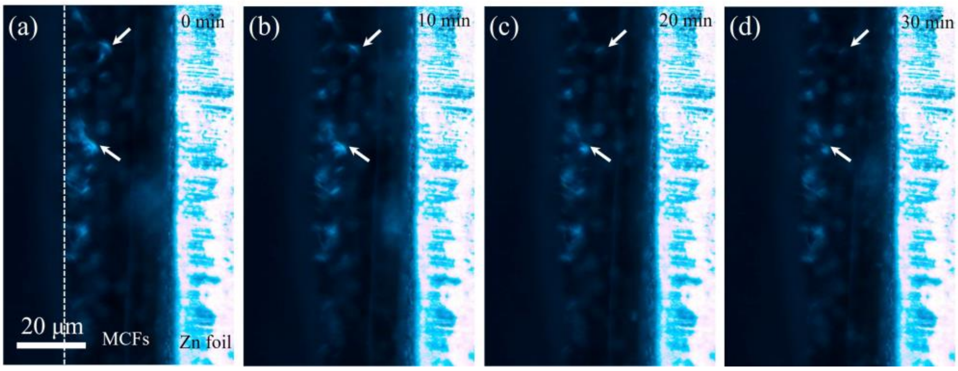

Fig. S19 In situ observation of stripping process of isolated Zn scattered on the surface of MCFs layer after 0, 10, 20, 30 mins at 1 mA cm^{-2}

Supporting Note: In order to confirm the "revival effect" of MCFs to the isolated Zn, some Zn particles were dispersed on the MCFs skeleton to simulate the isolated Zn produced during the plating/stripping cycle, and observed the morphology evolution of particles during the stripping process. As the in situ optical microscope observation shows, with the stripping process going on, the Zn particles scattered on the surface of MCFs layer dissolve gradually, indicating that the MCFs skeleton can build effective electrical contact between isolated Zn with the Zn foil. Of note, in practice, the possibly formed isolated Zn particles scattered in the fiber network, which is easier to form good electrical contact. As a result, the "dead Zn" can be effectively eliminated.

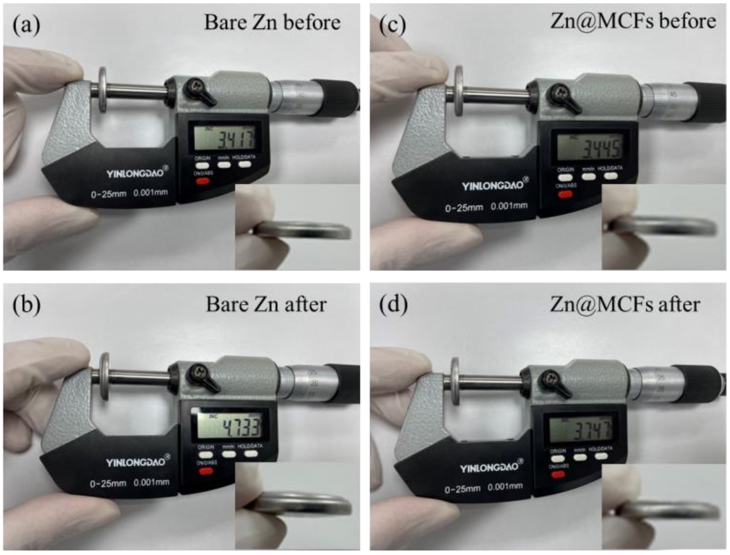

Fig. S20 Thickness of (**a**,**b**) bare Zn and (**c**,**d**) Zn@MCFs symmetric cells before and after plating/stripping test (1 mA cm^{-2})

Supporting Note: The bare Zn symmetric coin cell obviously swells after cycling, which results from the aerogenesis of the cells during cycling (Fig. S20a-b). The gas in the cells will increase the electrode-electrolyte interfacial impedance, thereby leading to the increasement in overpotential. Although the present of MCFs layer will inhibit the aerogenesis by reducing the overpotentials and stabilize the electrode-electrolyte interface, the slight bulge of the cell can still be observed after cycling (Fig. S20c-d). A higher overpotential is more likely to lead to hydrogen evolution, hence the fluctuation of overpotential in the late cycles is more likely to occur under the condition of a high rate and large deposition capacity.

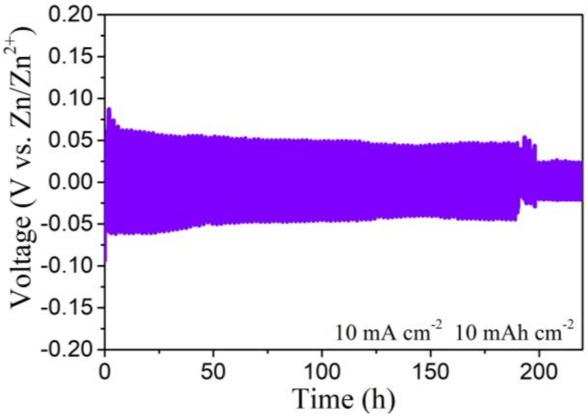

Fig. S21 Plating/stripping performance of Zn@MCFs symmetric battery with capacity of 10 mAh cm^{-2} at 10 mA cm^{-2}

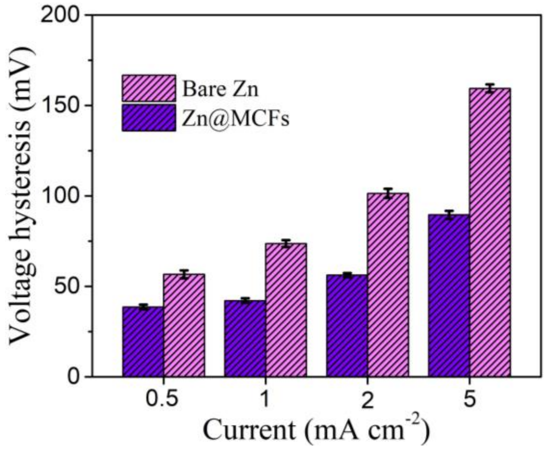

Fig. S22 The voltage hysteresis value comparison of bare Zn and Zn@MCFs symmetric cells at various current densities

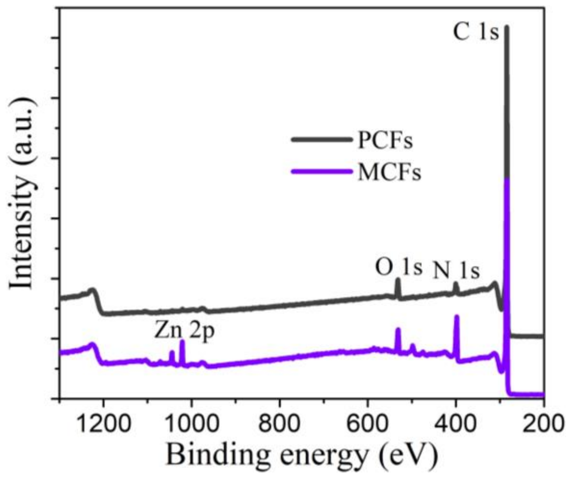

Fig. S23 XPS spectra of PCFs and MCFs

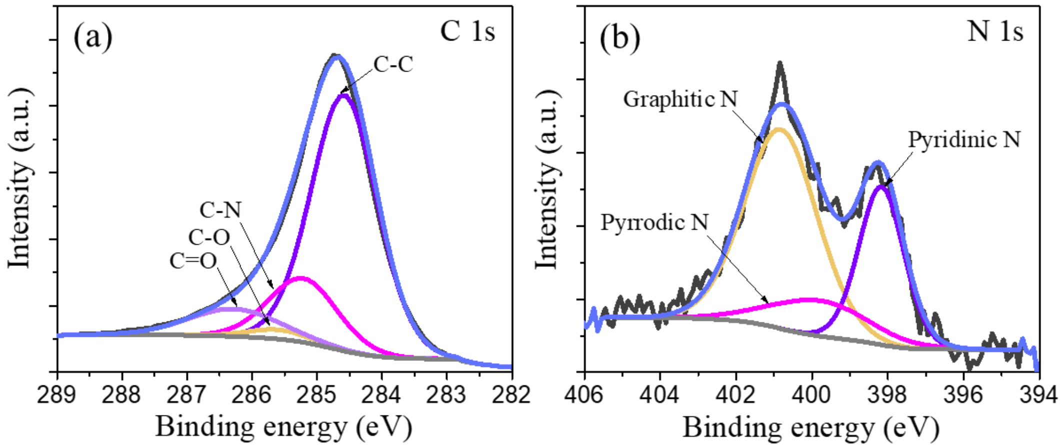

Fig. S24 XPS spectra and corresponding peak fitting result of (**a**) C 1s, and (**b**) N 1s for PCFs

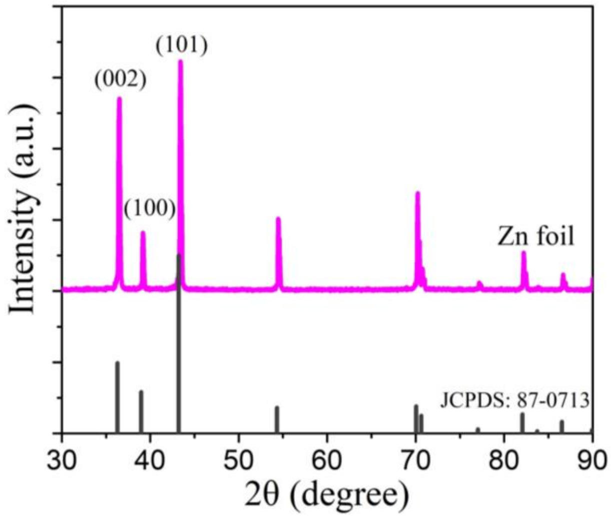

Fig. S25 XRD pattern of bare Zn foil

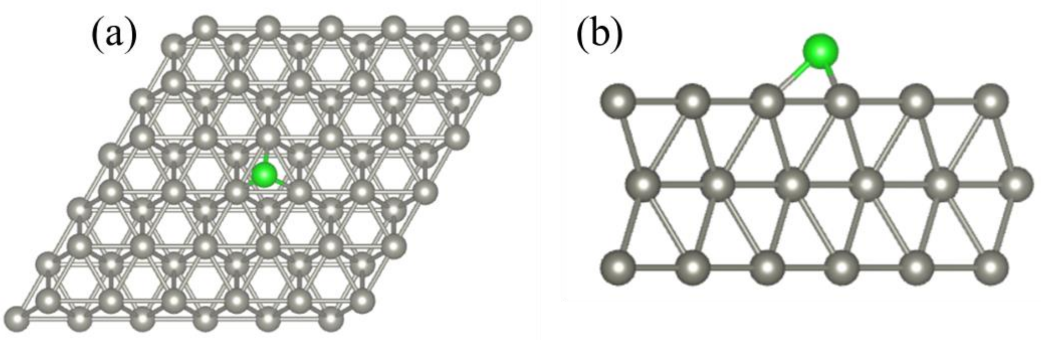

Fig. S26 Surface atomic configuration models of Zn (002): (**a**) Top view, and (**b**) side view

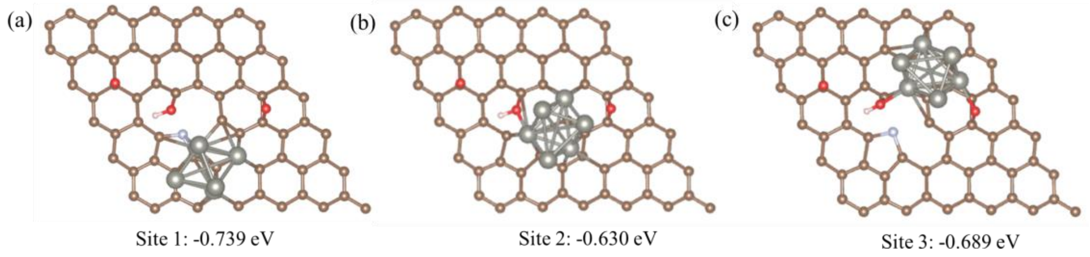

Fig. S27 (**a-c**) Binding energies of zinc clusters absorbed at different sites. Based on the calculation result, site 1 is selected as the surface atomic configuration model of MCFs

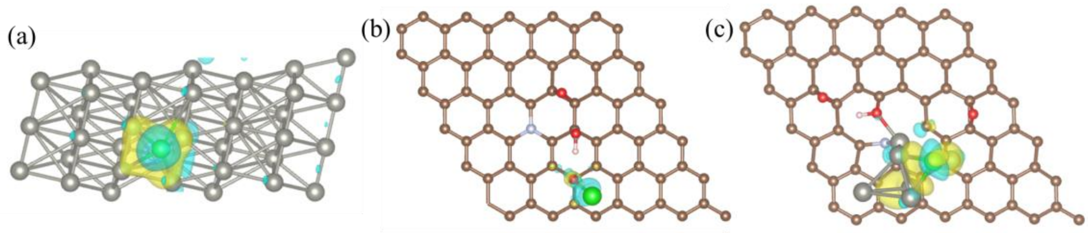

Fig. S28 Top view of electron clouds as zinc absorbed onto different substrates: (**a**) Zn (101), (**b**) PCFs and (**c**) MCFs

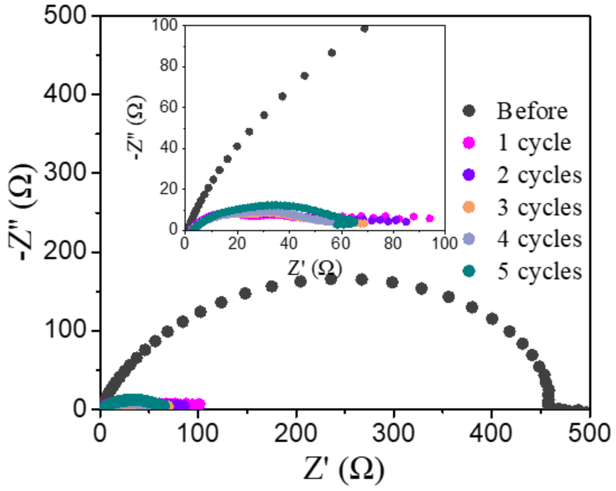

Fig. S29 Electrochemical impedances spectra of bare Zn symmetrical cells before and after 1, 2, 3, 4, 5 cycles, respectively

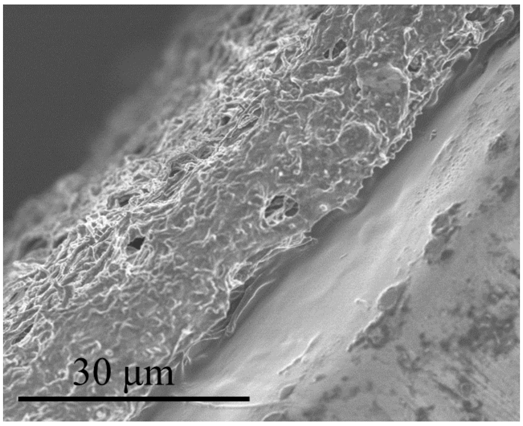

Fig. S30 Side view of the SEM image showing the interfacial morphology of deposited Zn@MCFs anode

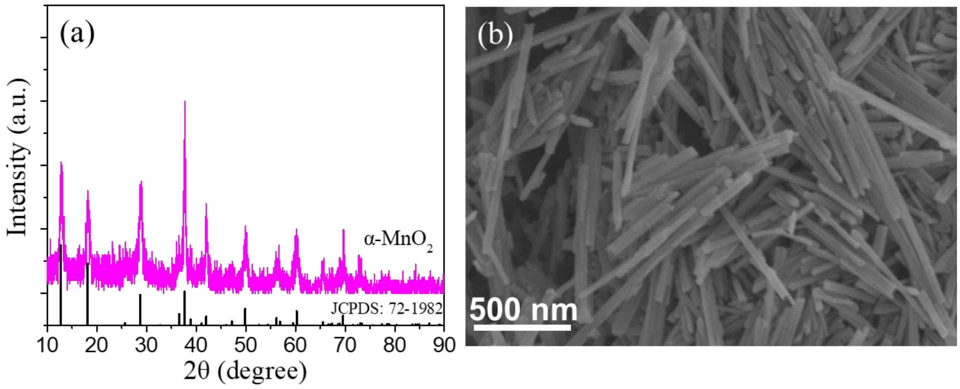

Fig. S31 (**a**) XRD pattern, and (**b**) SEM image of α-MnO_2 synthesized in lab

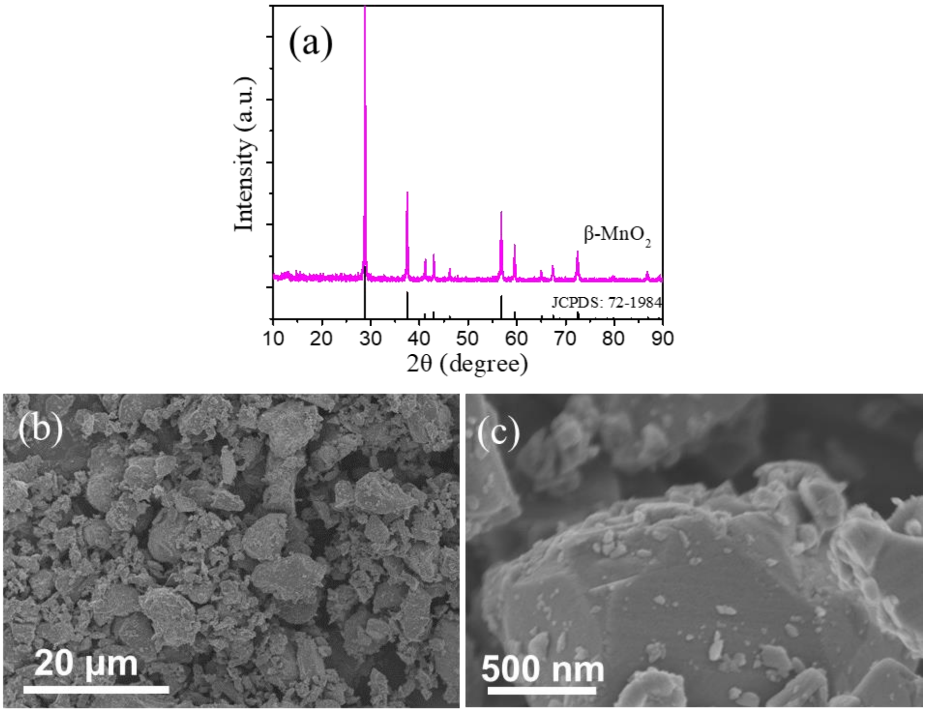

Fig. S32 (**a**) XRD pattern, and (**b**,**c**) SEM images of commercial β-MnO_2 purchased from Aladdin reagent (Shanghai) Co., LTD

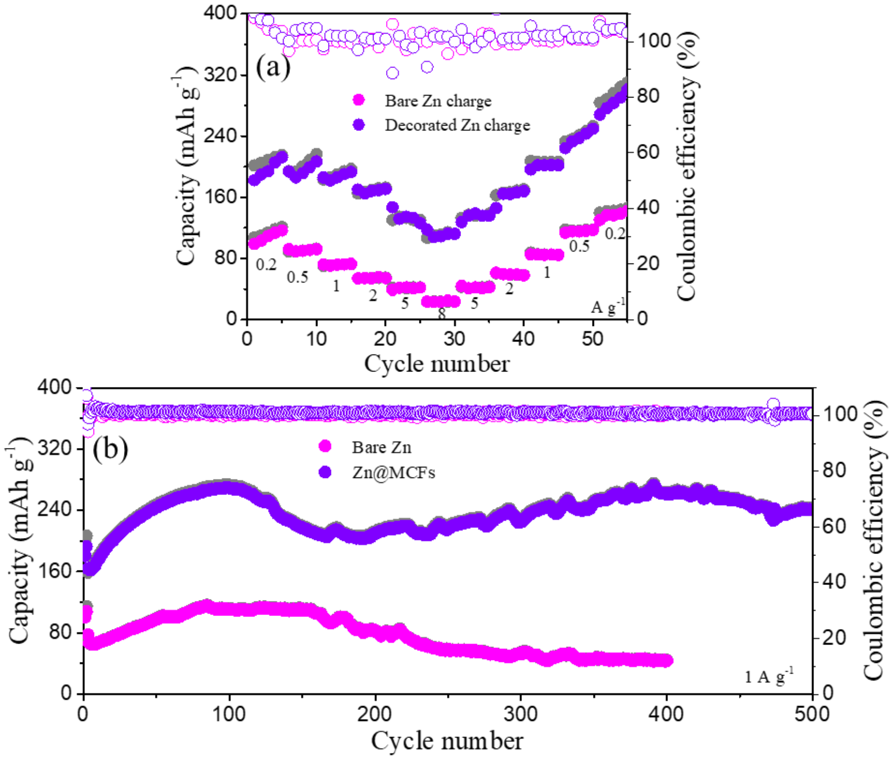

Fig. S33 Electrochemical performance of Zn||β-MnO_2 and Zn@MCFs||β-MnO_2 batteries. (**a**) Rate performance, and (**b**) cycling performance at 1 A g^{-1} (after activization at 0.2 A g^{-1} for two cycles)

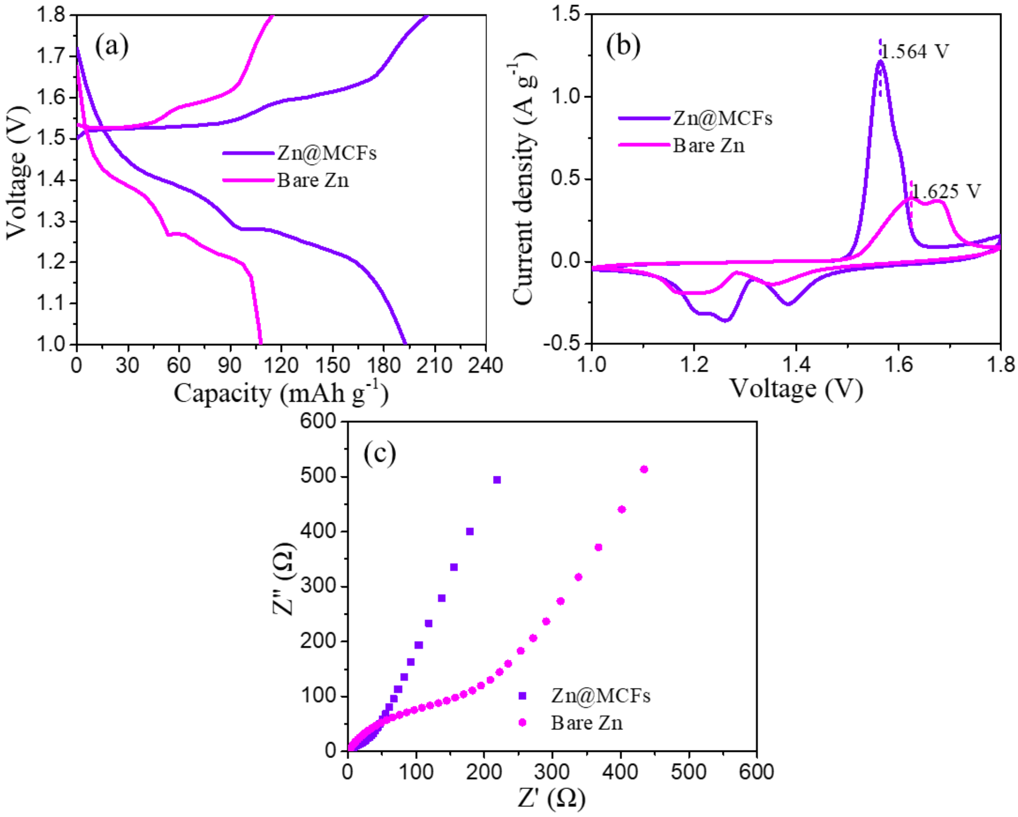

Fig. S34 Electrochemical performance of Zn||β-MnO_2 and Zn@MCFs||β-MnO_2 batteries. (**a**) Voltage-capacity curves at 0.2 A g^{-1}, (**b**) CV curves at 0.1 mV s^{-1}, and (**c**) EIS spectra before cycling

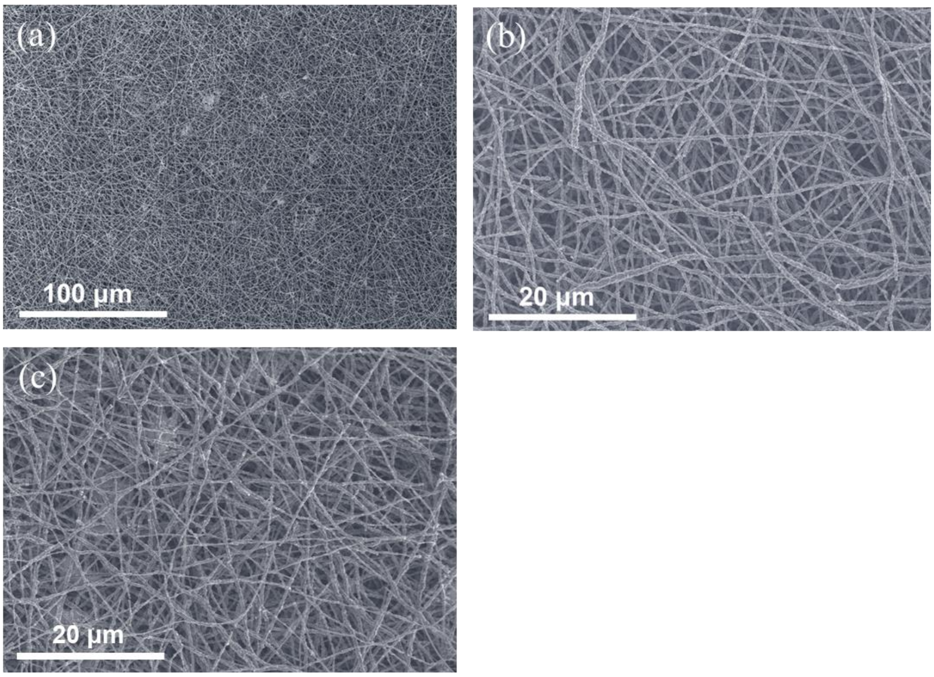

Fig. S35 SEM iamges of MCFs layer after test in (**a**,**b**) symmetric cell after 1500 h (5 mAh cm^{-2}, 1 mA cm^{-2}), and (**c**) Zn@MCFs||α-MnO_2 cell after 600 cycles at 1 A g^{-1}

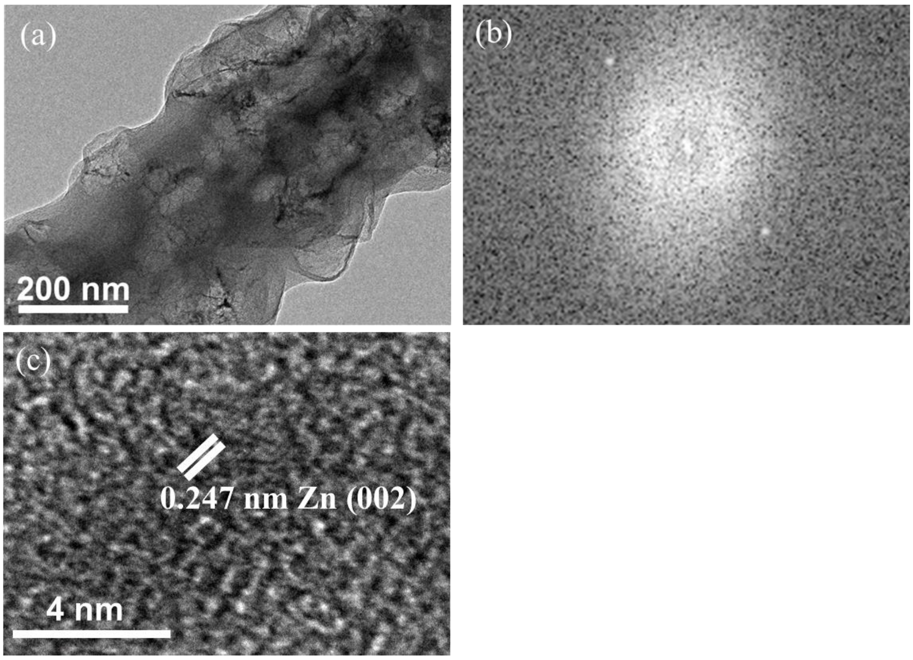

Fig. S36 (**a**) TEM, (**b**) FFT, and (**c**) HRTEM images of MCFs after 600 cycles in Zn@MCFs||α-MnO_2 cell

Supporting Note: In the current studies on aqueous Zn-ion batteries, excess zinc is usually used in the Zn-MnO_2 batteries, and the Zn anodes are operated at low depth of discharge (DOD). Hence, it is normal to detect small amounts of residual Zn nanocrystals in the nanofibers after cycles. Because of the excess zinc is used in the anode, the small amounts of residual zinc will not reduce the Coulomb efficiency. We also assessed the electrochemical performance of Zn@MCFs||MnO_2 pouch cell with controlled DOD (see below). Both coin cell with excess zinc and pouch cell with controlled DOD show high level of CEs, indicating the the Zn@MCFs anode and MnO_2 cathode show excellent electrochemical reversibility

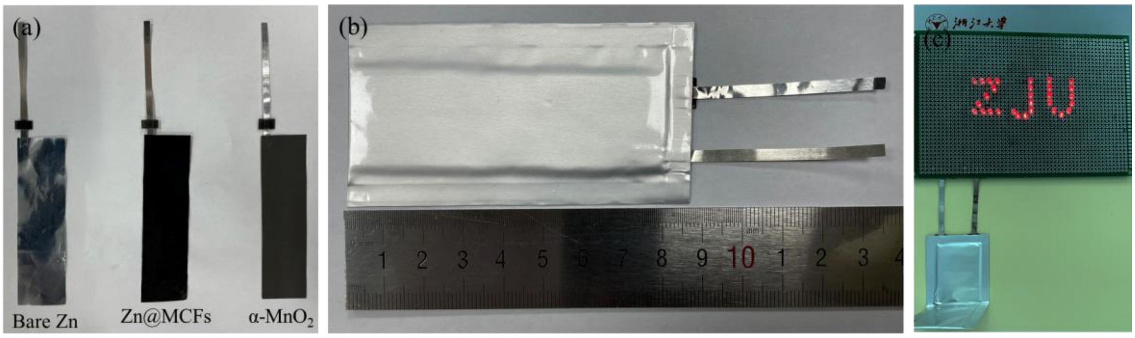

Fig. S37 Optical photographs of (**a**) bare Zn, Zn@MCFs, and α-MnO_2 electrodes, (**b**) pouch battery with Zn@MCFs anode and α-MnO_2 cathode (size: 2×8 cm^2), (**c**) digital photograph of 30 LEDs lit by the bent (90°) Zn@MCFs||α-MnO_2 pouch battery

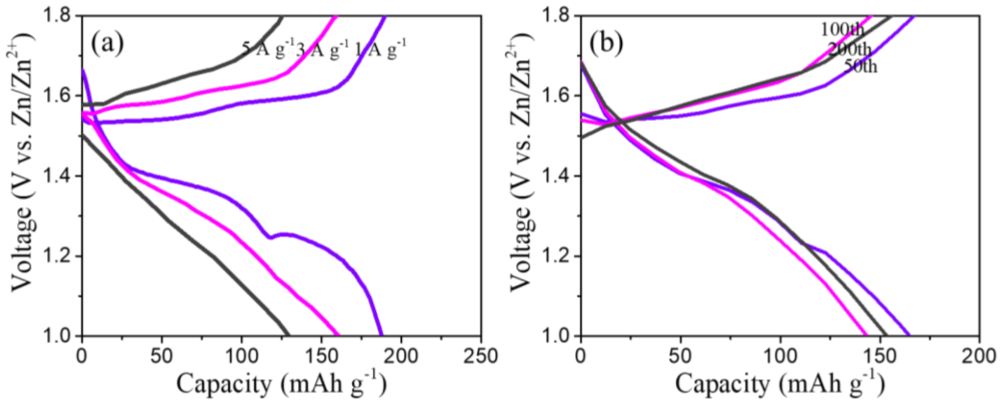

Fig. S38 Voltage-capacity curves of Zn@MCFs||α-MnO_2 pouch battery at (**a**) different current densities, and (**b**) different cycles

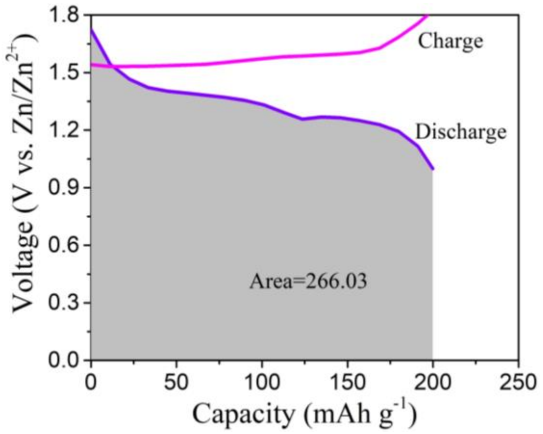

Fig. S39 Voltage-capacity curves of Zn@MCFs||α-MnO_2 pouch battery at 1 A g^{-1}, the specific discharge energy of $\int_0^{C_{discharge}}(C \times V)$ can be acquired according to the shaded area value

Table S1 Comparison of deposition capacity and cycle life of Zn anodes protected by various interfacial layers

Interfacial layer	Plating/stripping capacity (mAh cm^{-2})	Cycle life (h)	Refs.
ZIF-8	0.05	170	[S6]
3D nanoporous ZnO architecture	1.25	500	[S7]
Indium-based protective layer	0.05	1400	[S8]
MXene ($Ti_3C_2T_{x-y}$)	0.2	800	[S9]
Nanoporous $CaCO_3$	0.05	800	[S10]
Kaolin	1.1	800	[S11]
TiO_2	1	150	[S12]
3D interconnected ZnF_2	1	800	[S13]
ZnS	2	1100	[S14]
PVDF- $BaTiO_3$	2	1300	[S15]
Polyamide	0.25	8000	[S16]
Polyacrylonitrile	1	1100	[S17]
Cyanoacrylate adhesive	1	400	[S18]
Ag	0.2	1450	[S19]
In	0.2	1500	[S20]
Multicapsular carbon fibers	5	1500	This work

Supplementary References

[S1] M. Zhou, S. Guo, J. Li, X. Luo, Z. Liu et al., Surface-preferred crystal plane for a stable and reversible zinc anode. Adv. Mater. **33**(21), 2100187 (2021). https://doi.org/10.1002/adma.202100187

[S2] J. Zheng, Q. Zhao, T. Tang, J. Yin, C.D. Quilty et al., Reversible epitaxial electrodeposition of metals in battery anodes. Science **366**(6465), 645-648 (2019). https://doi.org/10.1126/science.aax6873

[S3] J. Cao, D. Zhang, C. Gu, X. Wang, S. Wang et al., Manipulating crystallographic orientation of zinc deposition for dendrite-free zinc ion batteries. Adv. Energy Mater. **11**(29), 2101299 (2021). https://doi.org/10.1002/aenm.202101299

[S4] Y. Hao, J. Zhou, G. Wei, A. liu, Y. Zhang et al., Artificial N-doped graphene protective layer enables stable Zn anode for aqueous Zn-ion batteries. ACS Appl. Energy Mater. **4**(6), 6364-6373 (2021). https://doi.org/10.1021/acsaem.1c01306

[S5] Y. Liang, Y. Wang, H. Mi, L. Sun, D. Ma et al., Functionalized carbon nanofiber interlayer towards dendrite-free, Zn-ion batteries. Chem. Eng. J. **425**, 131862 (2021). https://doi.org/10.1016/j.cej.2021.131862

[S6] X. Pu, B. Jiang, X. Wang, W. Liu, L. Dong et al., High-performance aqueous zinc-ion batteries realized by MOF materials. Nano-Micro Lett. **12**, 152 (2020). https://doi.org/10.1007/s40820-020-00487-1

[S7] X. Xie, S. Liang, J. Gao, S. Guo, J. Guo et al., Manipulating the ion-transfer kinetics and interface stability for high-performance zinc metal anodes. Energy Environ. Sci. **13**(2), 503-510 (2020). https://doi.org/10.1039/C9EE03545A

[S8] K. Hu, X. Guan, R. Lv, G. Li, Z. Hu et al., Stabilizing zinc metal anodes by artificial solid electrolyte interphase through a surface ion-exchanging strategy. Chem. Eng. J. **396**, 125363 (2020). https://doi.org/10.1016/j.cej.2020.125363

[S9] N. Zhang, S. Huang, Z. Yuan, J. Zhu, Z. Zhao et al., Direct self-assembly of MXene on Zn anodes for dendrite-free aqueous zinc-ion batteries. Angew. Chem. Int. Ed. **60**(6), 2861-2865 (2021). https://doi.org/10.1002/anie.202012322

[S10] L. Kang, M. Cui, F. Jiang, Y. Gao, H. Luo et al., Nanoporous $CaCO_3$ coatings enabled uniform Zn stripping/plating for long-life zinc rechargeable aqueous batteries. Adv. Energy Mater. **8**(25), 1801090 (2018). https://doi.org/10.1002/aenm.201801090

[S11] C. Deng, X. Xie, J. Han, Y. Tang, J. Gao et al., A sieve-functional and uniform-porous kaolin layer toward stable zinc metal anode. Adv. Funct. Mater. **30**(21), 2000599 (2020). https://doi.org/10.1002/adfm.202000599

[S12] K. Zhao, C. Wang, Y. Yu, M. Yan, Q. Wei et al., Ultrathin surface coating enables stabilized zinc metal anode. Adv. Mater. Interfaces **5**(16), 1800848 (2018). https://doi.org/10.1002/admi.201800848

[S13] Y. Yang, C. Liu, Z. Lv, H. Yang, Y. Zhang et al., Synergistic manipulation of Zn^{2+} ion flux and desolvation effect enabled by anodic growth of a 3D ZnF_2 matrix for long-lifespan and dendrite-free Zn metal anodes. Adv. Mater. **33**(11), 2007388 (2021). https://doi.org/10.1002/adma.202007388

[S14] J. Hao, B. Li, X. Li, X. Zeng, S. Zhang et al., An in-depth study of Zn metal surface chemistry for advanced aqueous Zn-ion batteries. Adv. Mater. **32**(34), 2003021 (2020). https://doi.org/10.1002/adma.202003021

[S15] P. Zou, R. Zhang, L. Yao, J. Qin, K. Kisslinger et al., Ultrahigh-rate and long-life

zinc–metal anodes enabled by self-accelerated cation migration. Adv. Energy Mater. **11**(31), 2100982 (2021). https://doi.org/10.1002/aenm.202100982

[S16] Z. Zhao, J. Zhao, Z. Hu, J. Li, J. Li et al., Long-life and deeply rechargeable aqueous Zn anodes enabled by a multifunctional brightener-inspired interphase. Energy Environ. Sci. **12**(6), 1938-1949 (2019). https://doi.org/10.1039/C9EE00596J

[S17] P. Chen, X. Yuan, Y. Xia, Y. Zhang, L. Fu et al., An artificial polyacrylonitrile coating layer confining zinc dendrite growth for highly reversible aqueous zinc-based batteries. Adv. Sci. **8**(11), 2100309 (2021). https://doi.org/10.1002/advs.202100309

[S18] Z. Cao, X. Zhu, D. Xu, P. Dong, M.O.L. Chee et al., Eliminating Zn dendrites by commercial cyanoacrylate adhesive for zinc ion battery. Energy Storage Mater. **36**, 132-138 (2021). https://doi.org/10.1016/j.ensm.2020.12.022

[S19] Q. Lu, C. Liu, Y. Du, X. Wang, L. Ding et al., Uniform Zn deposition achieved by Ag coating for improved aqueous zinc-ion batteries. ACS Appl. Mater. Interfaces **13**(14), 16869-16875 (2021). https://doi.org/10.1021/acsami.0c22911

[S20] D. Han, S. Wu, S. Zhang, Y. Deng, C. Cui et al., A corrosion-resistant and dendrite-free zinc metal anode in aqueous systems. Small **16**(29), 2001736 (2020). https://doi.org/10.1002/smll.202001736
